# Heritability and genome-wide association analyses of fasting plasma glucose in Chinese adult twins

**DOI:** 10.1186/s12864-020-06898-z

**Published:** 2020-07-18

**Authors:** Weijing Wang, Caixia Zhang, Hui Liu, Chunsheng Xu, Haiping Duan, Xiaocao Tian, Dongfeng Zhang

**Affiliations:** 1 Department of Epidemiology and Health Statistics, Public Health College, Qingdao University, No. 38 Dengzhou Road, Shibei District, Qingdao, 266021 Shandong Province China; 2 The First Hospital of Yulin, Yulin, Shanxi China; 3 Qingdao Municipal Center for Disease Control and Prevention, Qingdao, Shandong China; 4 Qingdao Institute of Preventive Medicine, Qingdao, Shandong China

**Keywords:** Fasting plasma glucose, Heritability, Genome-wide association study, Twins, Chinese

## Abstract

**Background:**

Currently, diabetes has become one of the leading causes of death worldwide. Fasting plasma glucose (FPG) levels that are higher than optimal, even if below the diagnostic threshold of diabetes, can also lead to increased morbidity and mortality. Here we intend to study the magnitude of the genetic influence on FPG variation by conducting structural equation modelling analysis and to further identify specific genetic variants potentially related to FPG levels by performing a genome-wide association study (GWAS) in Chinese twins.

**Results:**

The final sample included 382 twin pairs: 139 dizygotic (DZ) pairs and 243 monozygotic (MZ) pairs. The DZ twin correlation for the FPG level (r_DZ_ = 0.20, 95% CI: 0.04–0.36) was much lower than half that of the MZ twin correlation (r_MZ_ = 0.68, 95% CI: 0.62–0.74). For the variation in FPG level, the AE model was the better fitting model, with additive genetic parameters (A) accounting for 67.66% (95% CI: 60.50–73.62%) and unique environmental or residual parameters (E) accounting for 32.34% (95% CI: 26.38–39.55%), respectively. In the GWAS, although no genetic variants reached the genome-wide significance level (*P* < 5 × 10^− 8^), 28 SNPs exceeded the level of a suggestive association (*P* < 1 × 10^− 5^). One promising genetic region (2q33.1) around rs10931893 (*P* = 1.53 × 10^− 7^) was found. After imputing untyped SNPs, we found that rs60106404 (*P* = 2.38 × 10^− 8^) located at *SPATS2L* reached the genome-wide significance level, and 216 SNPs exceeded the level of a suggestive association. We found 1007 genes nominally associated with the FPG level (*P* < 0.05), including *SPATS2L*, *KCNK5*, *ADCY5*, *PCSK1*, *PTPRA*, and *SLC26A11*. Moreover, *C1orf74* (*P* = 0.014) and *SLC26A11* (*P* = 0.021) were differentially expressed between patients with impaired fasting glucose and healthy controls. Some important enriched biological pathways, such as *β*-alanine metabolism, regulation of insulin secretion, glucagon signaling in metabolic regulation, IL-1 receptor pathway, signaling by platelet derived growth factor, cysteine and methionine metabolism pathway, were identified.

**Conclusions:**

The FPG level is highly heritable in the Chinese population, and genetic variants are significantly involved in regulatory domains, functional genes and biological pathways that mediate FPG levels. This study provides important clues for further elucidating the molecular mechanism of glucose homeostasis and discovering new diagnostic biomarkers and therapeutic targets for diabetes.

## Background

Diabetes, as a chronic and metabolic disease, can cause serious damage to the blood vessels, heart, kidneys, nerves and eyes. This condition is one of the leading causes of death worldwide, and higher fasting plasma glucose (FPG) levels, even if below the diagnostic threshold of diabetes, can also lead to increased morbidity and mortality. Diabetes and higher-than-optimal FPG level together leaded to 3.7 million deaths from 1980 to 2014 worldwide [1]. Therefore, it is important to elucidate the underlying pathogenesis of increased FPG levels.

The FPG level is affected by both genetic and environmental factors. Currently, the magnitude of genetic impact on FPG variation has been researched in some studies. And the heritability of the FPG level varied, with 0–0.77 in Europeans [2–8], 0.16–0.51 in Americans [9–15] and 0.17–0.71 in Asians [16–22]. For the African population, two family studies found heritability values of 0.47 and 0.07 [23, 24]. Currently, genome-wide association studies (GWASs) are a promising approach to discover susceptibility genetic loci or genes associated with a phenotype. Several GWASs performed in Western countries found some genetic loci located at *ADCY5*, *G6PC2*, *MADD*, *TCF7L2*, *GCK*, *XIRP2*, *VPS16*, *PTPRA*, etc. [25–27]. However, few studies have explored the genetic effects on FPG levels in the Chinese population.

Chinese population are different from other ethnic populations in the aspect of genetic constitutions. Genetically related individuals (e.g. twins) will greatly increase the power of genetic association analysis and effectively identify the genetic variants potentially associated with complex traits [28]. Here, we performed this twin-based genetic epidemiological study to evaluate the magnitude of the genetic influence on FPG variation and further conducted a GWAS to identify specific genetic variants related to the FPG level in a sample of 382 Chinese twin pairs.

## Results

### Heritability

The final sample consisted of 382 twin pairs: 139 dizygotic (DZ) pairs and 243 monozygotic (MZ) pairs. The median (interquartile range) age for all twins was 50 (45–57) years, and the median (interquartile range) FPG level was 5.10 (4.59–5.80) mmol/L (Additional file 1).

After adjustment for the effect of covariates, the DZ twin correlation for the FPG level (r_DZ_ = 0.20, 95% CI: 0.04–0.36) was much lower than half of the MZ twin correlation (r_MZ_ = 0.68, 95% CI: 0.62–0.74), suggesting the genetic effect on the FPG level (Additional file 2).

As Table 1 shows, for the variation in FPG level, the AE model provided the better fit (AIC = 420.6, *P* > 0.05), with additive genetic parameters (A) accounting for 67.66% (95% CI: 60.50–73.62%) and unique environmental or residual parameters (E) accounting for 32.34% (95% CI: 26.38–39.55%), respectively.

**Table 1:** Model fit and proportion of variance for the FPG level accounted by genetic and environmental parameters

Model	Parameters estimates						Goodness of fit index				
	A% (95% CI)		D% (95% CI)		E% (95% CI)		-2LL	df	AIC	*χ*^2^	*P*
ADE	13.34	(0–70.30)	55.01	(0–73.91)	31.64	(25.99–38.51)	1933.448	757	419.4		
AE*	67.66	(60.50–73.62)	–	–	32.34	(26.38–39.55)	1936.625	758	420.6	0	1.00

**Note**: ^*^ the best fitted model, which was chosen on the basis of a change in *χ*^2^ not representing a significant worsening of fit

*FPG* fasting plasma glucose; *A* additive genetic effect; *D* common or shared environmental effect; *E* unique environmental or residual effect; *−2LL* −2 log likelihood; *df* degree of freedom; *AIC* Akaike’s information criterion; *χ*^*2*^, difference of *χ*^2^ value; *P*, *χ*^*2*^ test in model fitting

### GWAS

#### SNP-based analysis

The median age of 139 DZ twin pairs was 49 years (interquartile range: 45–56 years), and the median FPG level was 5.14 mmol/L (interquartile range: 4.60–5.90 mmol/L) (Additional file 1).

**Fig. 1:**
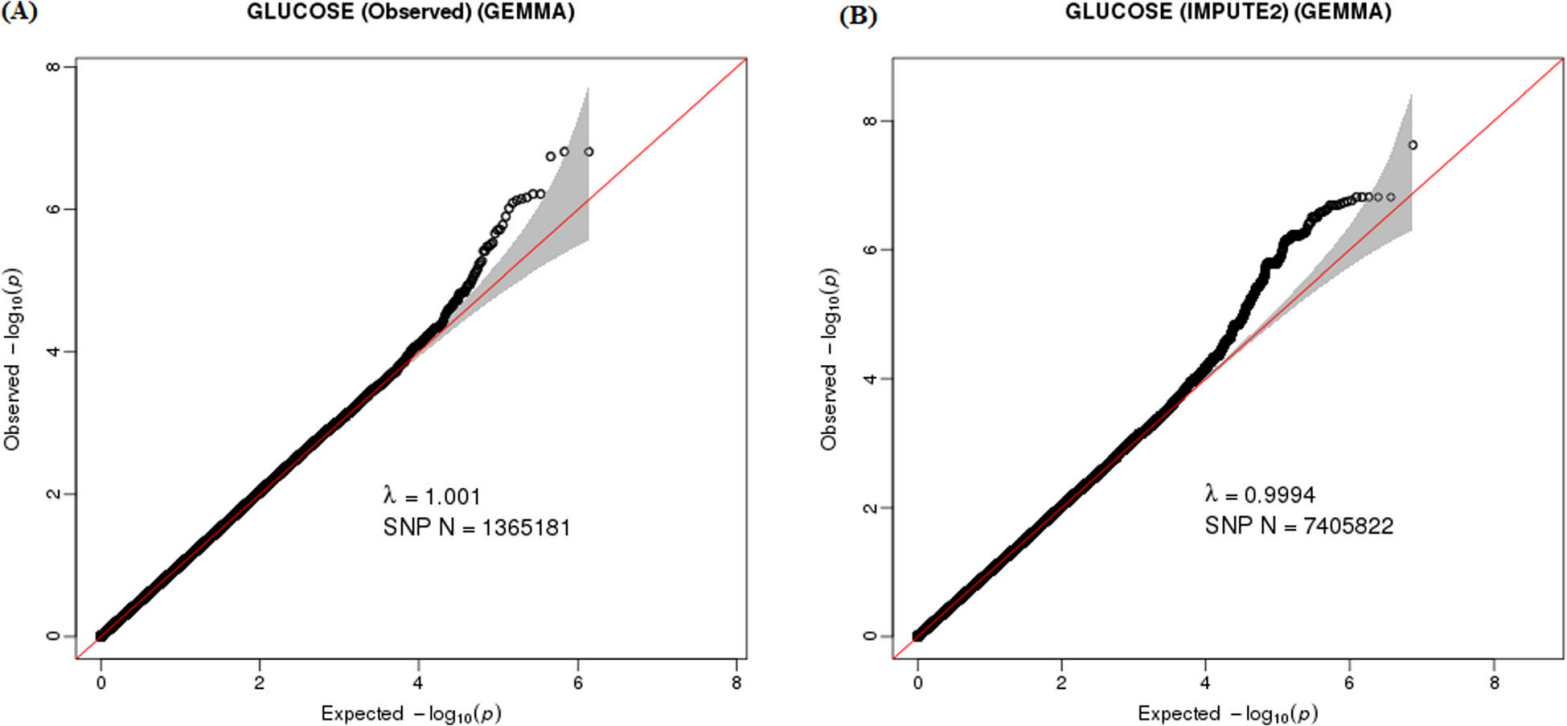
Quantile-quantile (Q-Q) plot for genome-wide association study (GWAS) of the fasting plasma glucose level. **a** The Q-Q plot of GWAS based on typed SNP data; **b** The Q-Q plot of GWAS based on imputed SNP data. The x-axis shows the -log_10_ of expected *P*-values of the association from the chi-square distribution, and the y-axis shows the -log_10_ of *P*-values from the observed chi-square distribution. The black dots represent the observed data with the top hit SNP being coloured, and the red line is the expectation under the null hypothesis of no association

**Fig. 2:**
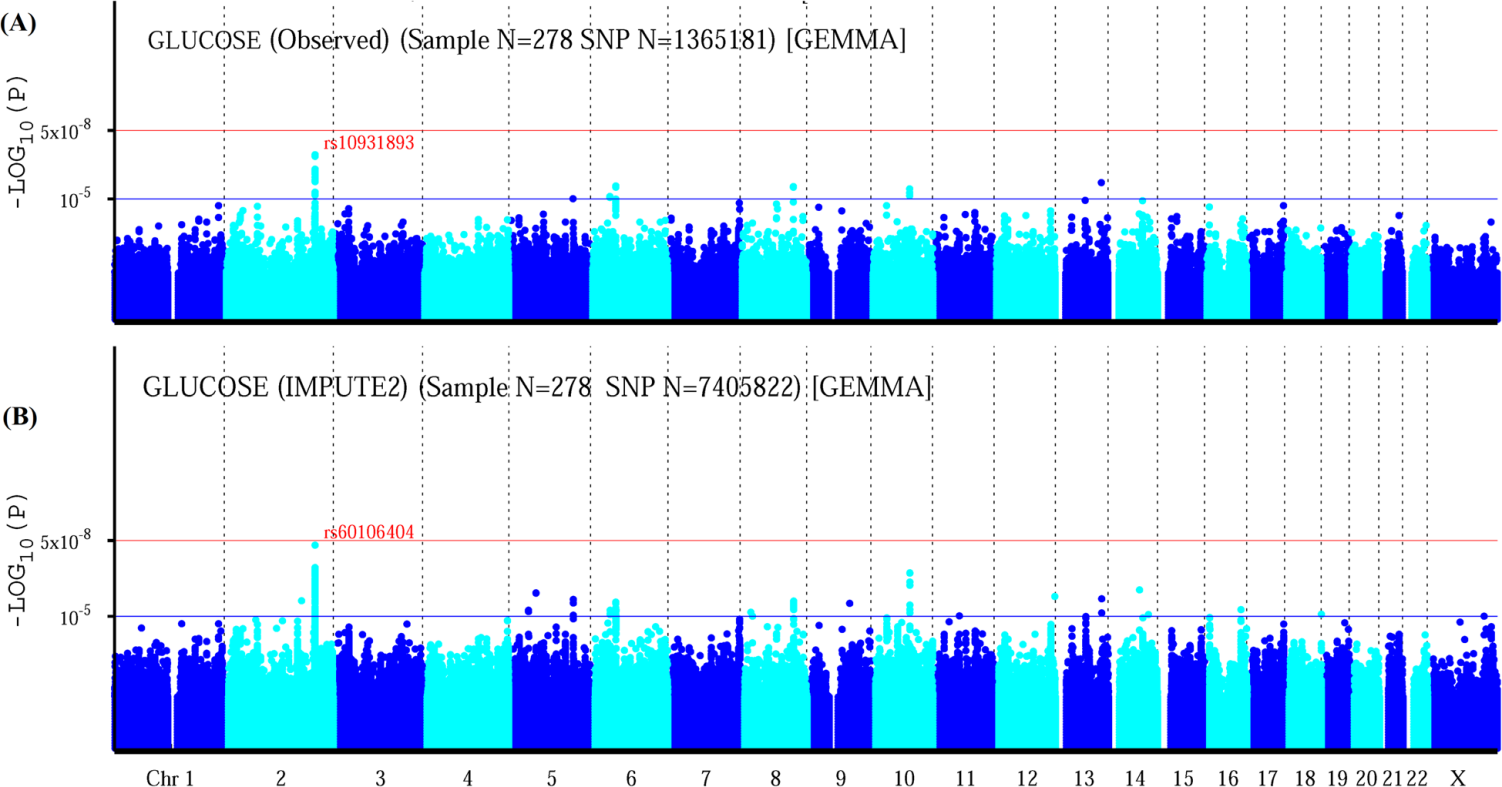
Manhattan plot for genome-wide association study (GWAS) of fasting plasma glucose level. **a** Manhattan plot of GWAS based on typed SNP data; **b** Manhattan plot of GWAS based on imputed SNP data. The x-axis shows the numbers of autosomes and the X chromosome, and the y-axis shows the -log_10_ of *P*-values for statistical significance. The dots represent the SNPs

The quantile-quantile (Q-Q) plot is shown in Fig. 1.a; there was no evidence of genomic inflation of test statistics or bias caused by population stratification (λ-statistic = 1.001). The slight deviation in the upper right tail from the null distribution indicated evidence of a weak association. None of the SNPs reached the genome-wide significance level (*P* < 5 × 10^− 8^), as illustrated by the Manhattan plot (Fig. 2.a). However, 28 SNPs were suggestive of association (*P* < 1 × 10^− 5^), with 17, 1, 4, 1, 4 and 1 SNPs located on chromosomes 2, 5, 6, 8, 10, and 13, respectively (Table 2). The strongest association was found with the SNP rs10931893 (*P* = 1.53 × 10^− 7^) on chromosome 2q33.1 at *SPATS2L*.

**Table 2:** Summary of genotyped SNPs (*P*-value < 1 × 10^− 5^) for association with the FPG level in genome-wide association study

SNP	Chr band	CHR	BP	***P***-value	Closest genes or genes	Official full name
rs10931893	2q33.1	2	201,114,652	1.53E-07	*SPATS2L*	Spermatogenesis associated serine rich 2 like
rs295134	2q33.1	2	201,110,223	1.53E-07	*SPATS2L*	Spermatogenesis associated serine rich 2 like
rs4516415	2q33.1	2	201,129,608	1.79E-07	*SPATS2L*	Spermatogenesis associated serine rich 2 like
rs295114	2q33.1	2	201,195,602	6.05E-07	*SPATS2L*	Spermatogenesis associated serine rich 2 like
rs1900706	2q33.1	2	201,214,071	6.05E-07	*SPATS2L*	Spermatogenesis associated serine rich 2 like
rs159320	2q33.1	2	201,187,775	6.79E-07	*SPATS2L*	Spermatogenesis associated serine rich 2 like
rs11691757	2q33.1	2	201,148,951	7.08E-07	*SPATS2L*	Spermatogenesis associated serine rich 2 like
rs10931896	2q33.1	2	201,148,076	7.43E-07	*SPATS2L*	Spermatogenesis associated serine rich 2 like
rs295118	2q33.1	2	201,144,004	8.08E-07	*SPATS2L*	Spermatogenesis associated serine rich 2 like
rs4673912	2q33.1	2	201,168,993	9.64E-07	*SPATS2L*	Spermatogenesis associated serine rich 2 like
rs295140	2q33.1	2	201,160,699	1.25E-06	*SPATS2L*	Spermatogenesis associated serine rich 2 like
rs10931897	2q33.1	2	201,162,520	1.64E-06	*SPATS2L*	Spermatogenesis associated serine rich 2 like
rs295129	2q33.1	2	201,229,473	1.91E-06	*SPATS2L*	Spermatogenesis associated serine rich 2 like
rs11890234	2q33.1	2	201,206,706	1.95E-06	*SPATS2L*	Spermatogenesis associated serine rich 2 like
rs10459299	13q32.3	13	99,776,084	2.17E-06	*DOCK9-AS2*	DOCK9 antisense RNA 2
rs9463802	6p12.2	6	52,469,904	2.93E-06	*TRAM2-AS1*	TRAM2 antisense RNA 1
rs6993473	8q23.3	8	116,054,890	3.09E-06	*LOC107986901*	Uncharacterized LOC107986901
rs3734434	6p12.2	6	52,460,604	3.29E-06	*TRAM2-AS1*	TRAM2 antisense RNA 1
rs11189019	10q23.1	10	83,018,925	3.83E-06	*RPA2P2*	Replication protein A2 pseudogene 2
rs10882870	10q23.1	10	83,019,949	3.83E-06	*RPA2P2*	Replication protein A2 pseudogene 2
rs1534599	2q33.1	2	201,073,133	5.28E-06	*SPATS2L*	Spermatogenesis associated serine rich 2 like
rs11188915	10q23.1	10	82,980,696	5.56E-06	*LOC105378386*	Uncharacterized LOC105378386
rs13035260	2q33.1	2	201,132,377	5.95E-06	*SPATS2L*	Spermatogenesis associated serine rich 2 like
rs10931890	2q33.1	2	201,102,055	6.94E-06	*SPATS2L*	Spermatogenesis associated serine rich 2 like
rs78736401	10q23.1	10	82,985,461	7.47E-06	*LOC105378386*	Uncharacterized LOC105378386
rs9470990	6p21.2	6	39,137,027	7.92E-06	*KCNK5*	Potassium two pore domain channel subfamily K member 5
rs10947785	6p21.2	6	39,132,818	8.57E-06	*KCNK5*	Potassium two pore domain channel subfamily K member 5
rs17097438	5q31.3	5	141,046,936	9.47E-06	*ARAP3*	ArfGAP with RhoGAP domain, ankyrin repeat and PH domain 3

**Note**: *BP* base pair; *CHR* chromosome

**Fig. 3:**
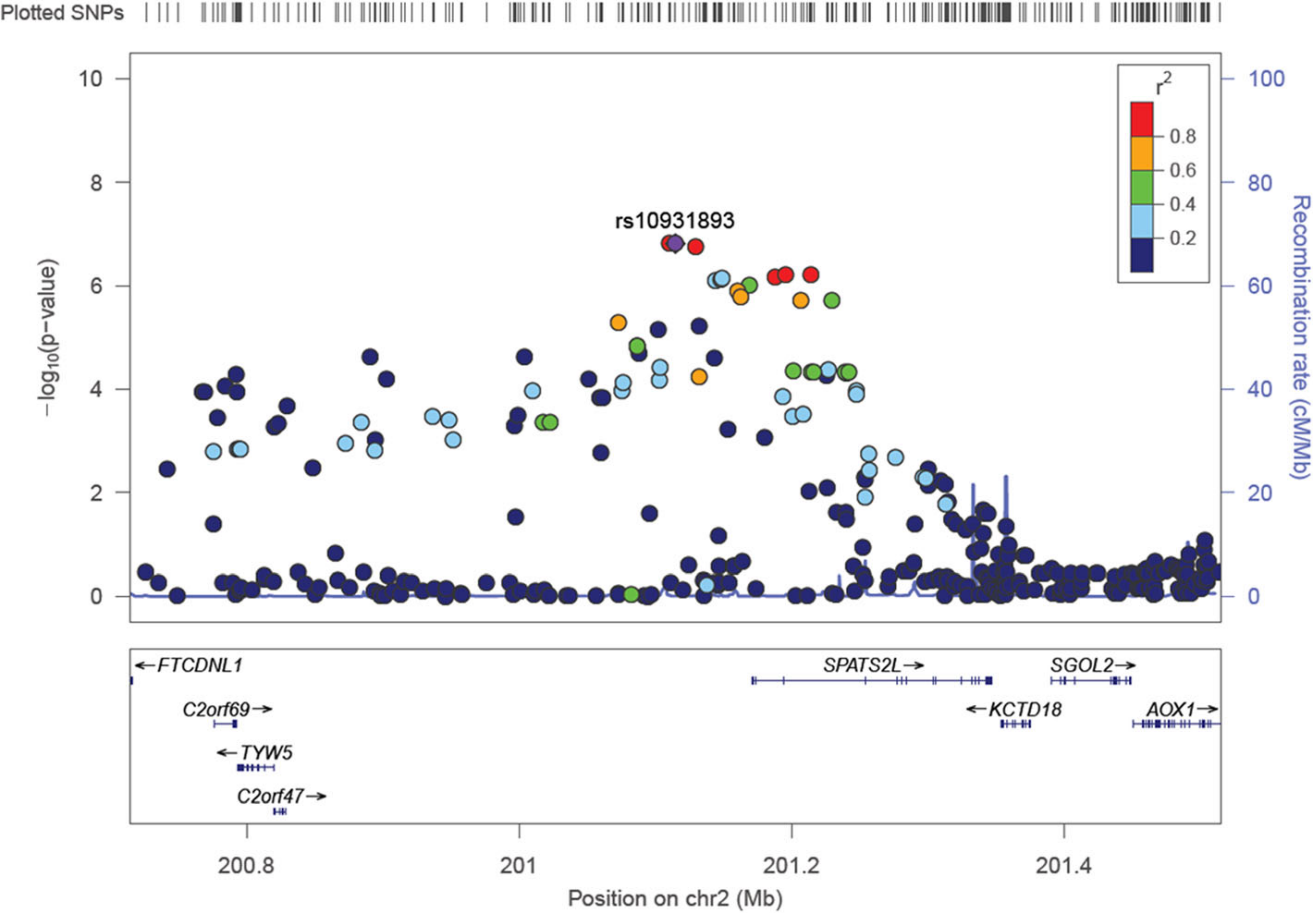
Regional association plot showing signals around chromosomal loci (2q33.1) for genome-wide association study of the fasting plasma glucose level

As shown in the regional association plot (Fig. 3), one promising chromosomal locus (2q33.1) around rs10931893 showed a potential association with FPG levels. In this region, 17 SNPs (*P* = 1.53 × 10^− 7^- 6.94 × 10^− 6^) were located at or close to *SPATS2L* which could moderate the protein expression of *β*_2_-adrenergic receptors [29]. Additionally, *SPATS2L* was nominally associated with FPG level (*P* < 0.05) in the subsequent gene-based analysis.

#### Post-imputation analysis

After performing imputation analysis, a total of 7,405,822 SNPs were identified for analysis. The Q-Q plot indicated evidence of moderate association (Fig. 1.b). One SNP, rs60106404 (*P* = 2.38 × 10^− 8^), located at *SPATS2L* reached the genome-wide significance level (*P* < 5 × 10^− 8^), as illustrated by the Manhattan plot (Fig. 2.b). A total of 216 SNPs showed suggestive evidence of an association (*P* < 1 × 10^− 5^) with the FPG level (Additional file 3).

When comparing our post-imputation results (*P* < 0.05) with previously reported FPG-associated SNPs found in relevant GWASs, we found that 8 SNPs rs7684538, rs2367204, rs7186570, rs861085, rs1402837, rs2302593, rs4869272 and rs492594 could be replicated (Additional file 4).

#### Gene-based analysis

Although none of the genes reached the genome-wide significance level (*P* < 2.63 × 10^− 6^), a total of 1007 genes were nominally associated with the FPG level (*P* < 0.05). The top 20 genes ranked by *P*-values are shown in Table 3. Several genes potentially related to FPG levels, including *BRAT1*, *TSPO*, *SLC2A12*, *KCNK5*, *PTPRA*, *ADCY5*, *PCSK1*, and *VPS16*, were found.

**Table 3:** The top 20 genes from gene-based analysis by using VEGAS2 tool

Chr	Gene	Number of SNPs	Start position	Stop position	Gene-based test statistic	***P-***value	Top SNP	Top SNP ***P-***value
2	SPATS2L^*^	92	201,170,603	201,346,986	543.69	1.20E-05	rs1900706	6.05E-07
7	*BRAT1*	11	2,577,443	2,595,392	95.24	4.00E-05	rs77213198	5.99E-05
12	*KNTC1*	15	123,011,808	123,110,947	170.32	4.80E-05	rs11058797	3.12E-05
2	*C2orf69*	7	200,775,978	200,792,996	60.26	8.70E-05	rs3098341	5.18E-05
17	*SGSH*	7	78,183,078	78,194,199	67.50	1.21E-04	rs7503034	2.50E-04
1	*C1orf74*	3	209,955,661	209,957,890	18.31	1.22E-04	rs7550857	2.39E-03
17	*CD300LF*	15	72,690,446	72,709,139	88.60	1.38E-04	rs2034310	2.09E-04
2	*SNRNP27*	2	70,121,074	70,132,368	21.37	2.04E-04	rs1048130	1.97E-05
14	*DPF3*	193	73,086,003	73,360,824	533.54	2.13E-04	rs12147969	1.14E-05
17	*RAB37*	36	72,667,255	72,743,474	166.45	2.19E-04	rs2034310	2.09E-04
22	*TSPO*	4	43,547,519	43,559,248	32.32	2.77E-04	rs138915	1.98E-04
3	*CLRN1*	38	150,643,949	150,690,786	166.19	2.87E-04	rs12497559	3.53E-04
19	*B9D2*	5	41,860,321	41,870,078	55.79	3.08E-04	rs11666933	1.47E-04
2	*TANK*	13	161,993,465	162,092,683	73.75	3.15E-04	rs57005826	4.51E-04
11	*OR4A16*	3	55,110,676	55,111,663	25.49	3.41E-04	rs10896659	2.76E-04
3	*GMNC*	13	190,570,525	190,580,465	59.08	4.69E-04	rs75145255	1.70E-03
2	*C2orf47*	2	200,820,039	200,828,847	24.29	4.89E-04	rs281767	4.46E-04
21	*LOC102724678*	13	39,698,280	39,717,998	69.17	5.91E-04	rs62218959	2.73E-04
12	*RSRC2*	7	122,989,189	123,011,560	57.31	6.06E-04	rs61956121	3.59E-04
17	*SLC26A11*	35	78,194,199	78,227,308	207.90	6.28E-04	rs4889999	1.89E-05

Note:^*^ Represented the genes had already been indicated in the SNP-based analysis

#### Pathway enrichment analysis

A total of 719 biological pathways were nominally associated with the FPG level (emp-*P* < 0.05) were found, and the top 30 pathways are shown in Table 4. The important pathways were mainly involved in *β*-alanine metabolism, regulation of insulin secretion, glucagon signaling in metabolic regulation, IL-1 receptor pathway, signaling by platelet derived growth factor (PDGF), cysteine and methionine metabolism, etc.

**Table 4:** The top 30 pathways (emp-*P* < 0.05) by using PASCAL tool

Pathway	chisq-***P***	emp-***P***	-log (chisq-***P***)	-log (emp-***P***)
BIOCARTA_G1_PATHWAY	1.02E-04	6.10E-06	3.9893	5.21467
KEGG_CELL_CYCLE	3.10E-04	1.36E-04	3.50818	3.86646
REACTOME_PYRIMIDINE_METABOLISM	1.26E-03	1.03E-03	2.90037	2.98716
REACTOME_PYRIMIDINE_CATABOLISM	1.26E-03	1.17E-03	2.90037	2.93181
KEGG_PANTOTHENATE_AND_COA_BIOSYNTHESIS	1.26E-03	1.42E-03	2.90037	2.84771
KEGG_BETA_ALANINE_METABOLISM	1.26E-03	1.37E-03	2.90037	2.86328
REACTOME_REGULATION_OF_INSULIN_SECRETION_BY_GLUCAGON_LIKE_PEPTIDE1	1.42E-03	1.73E-03	2.84744	2.76195
REACTOME_REGULATION_OF_INSULIN_SECRETION	1.64E-03	2.55E-03	2.78464	2.59346
BIOCARTA_P38MAPK_PATHWAY	1.83E-03	4.87E-04	2.7375	3.31247
REACTOME_GLUCAGON_SIGNALING_IN_METABOLIC_REGULATION	1.99E-03	2.10E-03	2.7012	2.67778
BIOCARTA_IL1R_PATHWAY	2.06E-03	5.20E-04	2.68591	3.284
REACTOME_G1_S_SPECIFIC_TRANSCRIPTION	2.11E-03	2.31E-03	2.67517	2.63639
BIOCARTA_SKP2E2F_PATHWAY	2.11E-03	1.94E-03	2.67517	2.7122
BIOCARTA_RACCYCD_PATHWAY	2.11E-03	1.90E-03	2.67517	2.72125
BIOCARTA_MCM_PATHWAY	2.11E-03	2.04E-03	2.67517	2.69037
KEGG_PYRIMIDINE_METABOLISM	2.12E-03	1.51E-03	2.67276	2.82102
REACTOME_SIGNALING_BY_PDGF	2.49E-03	2.37E-03	2.60427	2.62525
KEGG_TASTE_TRANSDUCTION	2.64E-03	3.83E-03	2.5779	2.4168
REACTOME_SYNTHESIS_OF_PIPS_AT_THE_PLASMA_MEMBRANE	2.69E-03	3.02E-03	2.56976	2.51999
BIOCARTA_FMLP_PATHWAY	2.69E-03	2.60E-03	2.56976	2.58503
BIOCARTA_NKT_PATHWAY	2.73E-03	9.20E-04	2.56384	3.03621
BIOCARTA_TOB1_PATHWAY	2.82E-03	2.87E-03	2.55023	2.54212
BIOCARTA_TGFB_PATHWAY	2.82E-03	3.01E-03	2.55023	2.52143
BIOCARTA_ALK_PATHWAY	2.82E-03	3.05E-03	2.55023	2.5157
KEGG_CYSTEINE_AND_METHIONINE_METABOLISM	2.85E-03	2.82E-03	2.54489	2.54975
REACTOME_E2F_MEDIATED_REGULATION_OF_DNA_REPLICATION	3.05E-03	1.78E-03	2.51614	2.74958
REACTOME_RORA_ACTIVATES_CIRCADIAN_EXPRESSION	3.06E-03	2.88E-03	2.5141	2.54061
REACTOME_CIRCADIAN_REPRESSION_OF_EXPRESSION_BY_REV_ERBA	3.06E-03	2.82E-03	2.5141	2.54975
BIOCARTA_RELA_PATHWAY	3.06E-03	2.99E-03	2.5141	2.52433
BIOCARTA_RARRXR_PATHWAY	3.06E-03	3.06E-03	2.5141	2.51428

#### Validation analysis

The gene expression levels of 25 genes in patients with impaired fasting glucose (IFG) and healthy controls (Additional file 5) were tested by the Wilcoxon rank sum method, and *C1orf74* (*P* = 0.014) and *SLC26A11* (*P* = 0.021) were differentially expressed between the two independent groups.

## Discussion

In this study, we evaluated the genetic contributions to FPG variation by twin modelling analyses and further identified the genetic variants associated with FPG levels by GWAS. We found that the heritability of FPG was 0.68, which was consistent with the previously reported range (0.22–0.71) in mainland China [16, 30–34].

Even no SNPs reached the genome-wide significance level, 28 SNPs showed suggestive evidence of an association with the FPG level. We found one promising genetic region (2q33.1) where 17 suggestive SNPs were linked to *SPATS2L*. *SPATS2L* might indirectly affect FPG levels by regulating the protein expression of *β*_2_-adrenergic receptors [29] that could increase glucose uptake [35, 36]. In addition, *SPATS2L* was the topmost gene in the gene-based analysis. Thus, *SPATS2L* may serve as candidate gene to be further validated and a potential biomarker for diabetes.

Post-imputation analysis revealed that one SNP, rs60106404, was significantly associated with the FPG level. This SNP is located at an important gene, *SPATS2L*, that has been discussed above. Furthermore, more than 200 SNPs were found to reach the level of a suggestive association. We compared our results with previously reported SNPs [25–27, 37–40] and found that 8 SNPs could be replicated, indicating our results were credible.

In the gene-based analysis, 1007 genes were nominally associated with FPG levels. Several interesting genes might influence FPG levels through the following mechanisms: (1) *BRAT1* deficiency could lead to increased glucose consumption [41]; (2) *TSPO* expression plays an important role in maintaining healthy adipocyte functions, and the activation of *TSPO* in adipocytes could improve glucose uptake [42]; (3) *SLC2A12*, a member of the solute carrier family, catalyzes the uptake of sugars through facilitated diffusion [43]; (4) the proteins encoded by *KCNK5* could influence the homeostasis of glucose by regulating insulin secretion [44]; (5) the protein encoded by the *PTPRA* gene is a member of the protein tyrosine phosphatase (PTP) family. *PTPRA* might play a role in insulin signaling as a negative regulator and further influence glucose homeostasis [45]. Moreover, the association between *PTPRA* and FPG levels has previously been reported [26]; (6) *ADCY5* plays a role in the normal regulation of insulin secretion [46], which might influence FPG levels. In addition, *ADCY5* has been previously reported to be associated with FPG levels [25, 27]; (7) the protein encoded by *PCSK1* is prohormone convertase 1/3 (PC1/3), which is essential to activate some peptide hormone precursors involved in regulating glucose homeostasis [47], and its association with FPG levels has also been previously reported [27]; (8) although the association of *VPS16* with FPG levels has been previously reported [26], its function in glucose metabolism is still unclear. However, other genes, especially the top 20 genes, were currently have unknown functions in glucose metabolism, and they may be potential candidate genes that need to be researched and validated in the future.

In addition, we tested the gene expression levels of several top genes in IFG cases and healthy controls, and found that *C1orf74* and *SLC26A11* were differentially expressed. *SLC26A11* was involved in the transport of glucose and other sugars, bile salts and organic acids, metal ions and amine compounds, as indicated by the GeneCards database, while the mechanism of *C1orf74* involved in blood glucose metabolism still needs to be explored.

The pathway enrichment analysis identified some important FPG-associated biological pathways: (1) *β*-alanine could significantly decrease glycolytic metabolism and change glycolytic-related gene expression [48]; (2) glucagon binding to its receptor could activate adenylate cyclase and improve cyclic adenosine monophosphate (cAMP) levels, which could promote insulin secretion [49–51]; (3) the IL-1R signaling system can regulate glucose homeostasis by sustaining the health and function of islet *β*-cells. When pancreatic IL-1R signaling is absent, the whole-body glucose homeostasis is disrupted [52]; (4) in the presence of sufficient PDGF receptor, PDGF can activate protein kinase B and result in the transportation of glucose transporter 4 (GLUT 4) to the surface of the cell, which finally promotes the absorption of glucose and produces an insulin-like effect [53–55]; (5) experimental and clinical studies have indicated that cysteine affects the regulation of insulin secretion and glucose levels. In addition, methionine could improve insulin sensitivity [56]; (6) PIPs can be phosphorylated by phosphatidylinositol 3-kinase to produce PIP3, which is involved in the insulin secretion signaling system by activating a PH-containing signaling protein such as protein kinase B [57, 58].

The strength of twin samples in our study was observed. The variation of human phenotype may be due to effects of genetic structure, gender, age and certain environmental exposures. Twin samples, as genetically related individuals, will highly increase the power of genetic association analysis and effectively find the genetic variants potentially associated with complex traits [28]. Hence, our results would be more credible.

Nevertheless, this study also has some limitations. This study was with a relatively small sample size because of the difficulties of recruiting and identifying qualified twin pairs. However, our results could still provide useful clues for hypotheses to be further replicated and validated while exploring the molecular mechanism of diabetes. Considering that the genetic influence on FPG variation is expected to be comprised of a lot of SNPs, a meta-analysis with a larger number of samples will be an ideal and desirable method.

## Conclusions

Our study has confirmed the significant contribution of genetic effects on FPG variation. The FPG level is highly heritable in the Chinese population, and some genetic variants are involved in regulatory domains, functional genes and biological pathways that mediate FPG levels. The results provide important clues for further elucidating the molecular mechanism of glucose homeostasis. The potential candidate biomarkers of FPG level presented here merit further verification.

## Materials and methods

The main materials and methods of this study were similar to our previously published studies [59, 60].

### Participants

Briefly, we collected samples through the Qingdao Twin Registry (QTR) [61, 62]. All twins took a questionnaire (Additional file 6) and underwent a health examination. We tested the FPG level by using the semiautomatic analyzer (Hitachi 7600, Japan). Twins who were pregnant or lactating, took hypoglycaemic agents, or used insulin were eliminated. We also dropped incomplete twin pairs. Finally, a total of 382 twin pairs (139 DZ pairs and 243 MZ pairs) aged 18 years or older were included in the heritability analysis and the subset of 139 DZ twin pairs was further included in the GWAS. All participants signed the written informed consent.

### Genotyping, quality control and imputation

Briefly, we firstly genotyped DNA samples, and then conducted quality control analysis [63]. 1,365,181 SNPs were obtained for subsequent SNP-based analysis. IMPUTE2 software [64] was used to impute untyped SNPs [65], and 7,405,822 SNPs were finally obtained.

### Heritability analysis

Genetic analysis were conducted by using Mx programme [66]. The FPG level was transformed following Blom’s formula for normality. Pearson’s product-moment correlation coefficient was calculated to measure intraclass phenotypic correlations per zygosity. When the correlation of DZ twins (r_DZ_) was much lower than half of that of MZ twins (r_MZ_), the ADE model was taken into account.

In the classical twin design, the phenotypic variation was decomposed into that due to additive genetic (A), dominant genetic (D), and unique environmental or residual (E) influences. Standard structural equation modeling (SEM) methods were used to estimate the A, D, and E components while adjusting for age, sex, and BMI. We performed a likelihood ratio test to compare the performances of the full ADE model and its nested model, i.e., AE model. And the better fitting model was chosen according to the parsimonious principle and a lower Akaike’s information criterion (AIC) value [67]. The power of this classical twin design was above 90%, which was computed based on the sample size combining significance level *α* and degree of freedom by the Mx programme.

### GWAS

#### SNP-based analysis

GEMMA [63] was adopted to evaluate the association of FPG levels with SNP genotypes with adjusting for age, sex, and BMI. The conventional Bonferroni-corrected threshold (5 × 10^− 8^) was set as genome-wide statistical significance, and the commonly used threshold (1 × 10^− 5^) was adopted for suggestive hits [68–70].

#### Gene-based analysis

We performed this analysis by using VEGAS2 tool [71, 72]. The genes showing more signal or strength of association than expected by chance were found. *P* < 2.63 × 10^− 6^ was set as genome-wide statistical significance.

#### Pathway enrichment analysis

We used the PASCAL tool to compute the gene and pathway scores [73]. Individual SNPs from GWAS were firstly mapped to genes involved in each pathway. The default parameter values were employed, including all markers inside the gene ±50 flanking kb and the maximum number of 3000 SNPs per gene. Then the gene scores for all genes in one pathway were computed and a joint score was estimated. At last, the pathway scores were computed, and the pathway enrichment of high-scoring genes was evaluated through two parameter-free procedures, e.g., chi-square and empirical scores. BioCarta, KEGG and Reactome were selected in the MSigDB database to obtain pathways and corresponding gene annotation [74].

#### Validation analysis

The blood samples of 8 subjects (4 cases and 4 healthy controls) were sequenced to obtain the gene expression data. The cases were defined as those with FPG ≥ 6.1 mmol/L, i.e., IFG status, and the healthy controls were defined as those with FPG ≤ 4.7 mmol/L. Then, the expression levels of 25 genes, i.e., the genes where the top SNPs were located and the top 20 genes in the VEGAS2 analysis, between the two independent groups were compared by the Wilcoxon rank sum test. We set the *P*-value < 0.05 as statistically significant.

## Supplementary information

**Additional file 1. ** Descriptive statistics for twins.

**Additional file 2. ** Phenotypic correlation coefficients (95% confidence intervals) with effects of covariates in twin pairs.

**Additional file 3. **Summary of the imputed SNPs with a *P*-value < 1 × 10^− 5^ for association of the FPG level in GWAS.

**Additional file 4. **Comparison between our imputed SNPs (*P*-value < 0.05) and other previously reported SNPs in GWAS.

**Additional file 5.** The characteristics of subjects in validation analysis.

**Additional file 6.** The questionare of survey on health status of elderly twins in Qingdao city.

## Data Availability

The dataset supporting the conclusions of this article is available in the European Variation Archive (EVA) repository (Accession No. PRJEB23749).

## Abbreviations

DZ: Dizygotic
FPG: Fasting plasma glucose
GWAS: Genome-wide association study
MZ: Monozygotic
