# Supplementary material for: Heritability and genome-wide association analyses of fasting plasma glucose in Chinese adult twins
Source: BMC Genomics. 2020 Jul 18;21:491. doi: 10.1186/s12864-020-06898-z (PMC7368793; doi:10.1186/s12864-020-06898-z)
Supplement: Supplementary file 1 — Additional file 1. Descriptive statistics for twins. [file 12864_2020_6898_MOESM1_ESM.docx]

**Additional file 1.** Descriptive statistics for twins

| **Subjects** | **Variable** | **Male** | | | **Female** | | | **ALL** | | |
| --- | --- | --- | --- | --- | --- | --- | --- | --- | --- | --- |
|  |  | N | Range (minimum-maximum) | Median (interquartile range) | N | Range (minimum-maximum) | Median (interquartile range) | N | Range (minimum-maximum) | Median (interquartile range) |
| ALL  sample | Age (years) | 371 | 33-80 | 50  (45-58) | 393 | 39-70 | 50  (46-55.5) | 764 | 33-80 | 50  (45-57) |
|  | FPG (mmol/L) | 371 | 3.10-15.50 | 5.40  (4.80-6.10) | 393 | 3.00-21.10 | 4.84 (4.41-5.48) | 764 | 3.00-21.10 | 5.10 (4.59-5.80) |
| GWAS  sample | Age (years) | 141 | 41-70 | 50  (45-56) | 137 | 40-70 | 49  (45-56) | 278 | 40-70 | 49  (45-56) |
|  | FPG (mmol/L) | 141 | 3.50-10.44 | 5.30  (4.79-6.10) | 137 | 3.70-21.10 | 4.98 (4.53-5.64) | 278 | 3.50-21.10 | 5.14 (4.60-5.90) |

**Note**: FPG, fasting plasma glucose
