# Supplementary material for: Heritability and genome-wide association analyses of fasting plasma glucose in Chinese adult twins
Source: BMC Genomics. 2020 Jul 18;21:491. doi: 10.1186/s12864-020-06898-z (PMC7368793; doi:10.1186/s12864-020-06898-z)
Supplement: Supplementary file 2 — Additional file 2. Phenotypic correlation coefficients (95% confidence intervals) with effects of covariates in twin pairs. [file 12864_2020_6898_MOESM2_ESM.docx]

**Additional file 2.** Phenotypic correlation coefficients (95% confidence intervals) with effects of covariates in twin pairs

| **Model** | **MZ** | | **DZ** | | ***-2LL*** | ***df*** | ***χ^2^*** | ***P*-value** |
| --- | --- | --- | --- | --- | --- | --- | --- | --- |
|  | cor. | (95%CI) | cor. | (95%CI) |  |  |  |  |
| Base | 0.684 | (0.615-0.740) | 0.204 | (0.038-0.355) | 1933.4 | 757 | - | - |
| No sex | 0.697 | (0.631-0.751) | 0.236 | (0.067-0.385) | 1956.4 | 758 | 22.9 | <0.001 |
| No age | 0.684 | (0.615-0.740) | 0.203 | (0.037-0.354) | 1933.9 | 758 | 0.5 | 0.479 |
| No BMI | 0.692 | (0.625-0.747) | 0.226 | (0.062-0.373) | 1944.8 | 758 | 11.3 | <0.001 |

**Note**: MZ, monozygotic; DZ, dizygotic; -2LL, -2 log likelihood; df, degree of freedom; *χ^2^*, difference of *χ^2^* value; corr., correlation coefficient; BMI, body mass index
