# Supplementary material for: Heritability and genome-wide association analyses of fasting plasma glucose in Chinese adult twins
Source: BMC Genomics. 2020 Jul 18;21:491. doi: 10.1186/s12864-020-06898-z (PMC7368793; doi:10.1186/s12864-020-06898-z)
Supplement: Supplementary file 3 — Additional file 3. Summary of the imputed SNPs with a P-value < 1 × 10− 5 for association of the FPG level in GWAS. [file 12864_2020_6898_MOESM3_ESM.docx]

**Additional file 3.** Summary of the imputed SNPs with a *P*-value < 1×10^-5^ for association of the FPG level in GWAS

| **SNP** | **Chr band** | **CHR** | **BP** | ***P*-value** | **Closest genes or genes** | **Official full name** |
| --- | --- | --- | --- | --- | --- | --- |
| rs60106404 | 2q33.1 | 2 | 201118971 | 2.38227E-08 | *SPATS2L* | Spermatogenesis associated serine rich 2 like |
| rs295119 | 2q33.1 | 2 | 201117944 | 1.53405E-07 | *SPATS2L* | Spermatogenesis associated serine rich 2 like |
| rs10931893 | 2q33.1 | 2 | 201114652 | 1.53405E-07 | *SPATS2L* | Spermatogenesis associated serine rich 2 like |
| rs295134 | 2q33.1 | 2 | 201110223 | 1.53405E-07 | *SPATS2L* | Spermatogenesis associated serine rich 2 like |
| rs1369842 | 2q33.1 | 2 | 201108987 | 1.53405E-07 | *SPATS2L* | Spermatogenesis associated serine rich 2 like |
| rs4673814 | 2q33.1 | 2 | 201108133 | 1.53405E-07 | *SPATS2L* | Spermatogenesis associated serine rich 2 like |
| rs10804097 | 2q33.1 | 2 | 201104924 | 1.73678E-07 | *SPATS2L* | Spermatogenesis associated serine rich 2 like |
| rs4516415 | 2q33.1 | 2 | 201129608 | 1.79096E-07 | *SPATS2L* | Spermatogenesis associated serine rich 2 like |
| rs4673944 | 2q33.1 | 2 | 201198471 | 1.8643E-07 | *SPATS2L* | Spermatogenesis associated serine rich 2 like |
| rs34467224 | 2q33.1 | 2 | 201133705 | 1.96445E-07 | *SPATS2L* | Spermatogenesis associated serine rich 2 like |
| rs12474914 | 2q33.1 | 2 | 201130210 | 2.04603E-07 | *SPATS2L* | Spermatogenesis associated serine rich 2 like |
| rs4233994 | 2q33.1 | 2 | 201129211 | 2.04603E-07 | *SPATS2L* | Spermatogenesis associated serine rich 2 like |
| rs12469091 | 2q33.1 | 2 | 201124735 | 2.04603E-07 | *SPATS2L* | Spermatogenesis associated serine rich 2 like |
| rs13007517 | 2q33.1 | 2 | 201129729 | 2.04603E-07 | *SPATS2L* | Spermatogenesis associated serine rich 2 like |
| rs10931892 | 2q33.1 | 2 | 201104997 | 2.3108E-07 | *SPATS2L* | Spermatogenesis associated serine rich 2 like |
| rs10882871 | 10q23.1 | 10 | 83021933 | 2.44823E-07 | *RPA2P2* | Replication protein A2 pseudogene 2 |
| rs842830 | 2q33.1 | 2 | 201131124 | 2.53713E-07 | *SPATS2L* | Spermatogenesis associated serine rich 2 like |
| rs4233996 | 2q33.1 | 2 | 201131228 | 2.62753E-07 | *SPATS2L* | Spermatogenesis associated serine rich 2 like |
| rs4673854 | 2q33.1 | 2 | 201134736 | 2.64252E-07 | *SPATS2L* | Spermatogenesis associated serine rich 2 like |
| rs3036485 | 2q33.1 | 2 | 201194504 | 2.74868E-07 | *SPATS2L* | Spermatogenesis associated serine rich 2 like |
| rs1217429 | 2q33.1 | 2 | 201094151 | 3.14944E-07 | *SPATS2L* | Spermatogenesis associated serine rich 2 like |
| rs1217456 | 2q33.1 | 2 | 201092279 | 3.14944E-07 | *SPATS2L* | Spermatogenesis associated serine rich 2 like |
| rs1729421 | 2q33.1 | 2 | 201080113 | 3.15053E-07 | *SPATS2L* | Spermatogenesis associated serine rich 2 like |
| rs952530 | 2q33.1 | 2 | 201081745 | 3.15053E-07 | *SPATS2L* | Spermatogenesis associated serine rich 2 like |
| rs13022984 | 2q33.1 | 2 | 201116067 | 3.15657E-07 | *SPATS2L* | Spermatogenesis associated serine rich 2 like |
| rs4673855 | 2q33.1 | 2 | 201136143 | 3.86457E-07 | *SPATS2L* | Spermatogenesis associated serine rich 2 like |
| rs59270066 | 2q33.1 | 2 | 201135026 | 3.86457E-07 | *SPATS2L* | Spermatogenesis associated serine rich 2 like |
| rs13019821 | 2q33.1 | 2 | 201106815 | 4.10583E-07 | *SPATS2L* | Spermatogenesis associated serine rich 2 like |
| rs13035260 | 2q33.1 | 2 | 201132377 | 4.65441E-07 | *SPATS2L* | Spermatogenesis associated serine rich 2 like |
| rs11189031 | 10q23.1 | 10 | 83023100 | 5.28275E-07 | *RPA2P2* | Replication protein A2 pseudogene 2 |
| rs4145969 | 2q33.1 | 2 | 201139961 | 5.47259E-07 | *SPATS2L* | Spermatogenesis associated serine rich 2 like |
| rs2060122 | 2q33.1 | 2 | 201153177 | 5.5275E-07 | *SPATS2L* | Spermatogenesis associated serine rich 2 like |
| rs4673837 | 2q33.1 | 2 | 201131159 | 5.60825E-07 | *SPATS2L* | Spermatogenesis associated serine rich 2 like |
| rs1729412 | 2q33.1 | 2 | 201083598 | 5.66078E-07 | *SPATS2L* | Spermatogenesis associated serine rich 2 like |
| rs149709064 | 2q33.1 | 2 | 201115134 | 5.91724E-07 | *SPATS2L* | Spermatogenesis associated serine rich 2 like |
| rs1436164 | 2q33.1 | 2 | 201158411 | 5.91876E-07 | *SPATS2L* | Spermatogenesis associated serine rich 2 like |
| rs3739119 | 2q33.1 | 2 | 201194279 | 6.04536E-07 | *SPATS2L* | Spermatogenesis associated serine rich 2 like |
| rs296818 | 2q33.1 | 2 | 201176944 | 6.04536E-07 | *SPATS2L* | Spermatogenesis associated serine rich 2 like |
| rs295137 | 2q33.1 | 2 | 201150040 | 6.04536E-07 | *SPATS2L* | Spermatogenesis associated serine rich 2 like |
| rs3769471 | 2q33.1 | 2 | 201195403 | 6.04536E-07 | *SPATS2L* | Spermatogenesis associated serine rich 2 like |
| rs295114 | 2q33.1 | 2 | 201195602 | 6.04536E-07 | *SPATS2L* | Spermatogenesis associated serine rich 2 like |
| rs295141 | 2q33.1 | 2 | 201163556 | 6.04536E-07 | *SPATS2L* | Spermatogenesis associated serine rich 2 like |
| rs295113 | 2q33.1 | 2 | 201197816 | 6.04536E-07 | *SPATS2L* | Spermatogenesis associated serine rich 2 like |
| rs295149 | 2q33.1 | 2 | 201175244 | 6.04536E-07 | *SPATS2L* | Spermatogenesis associated serine rich 2 like |
| rs1900706 | 2q33.1 | 2 | 201214071 | 6.04536E-07 | *SPATS2L* | Spermatogenesis associated serine rich 2 like |
| rs5837730 | 2q33.1 | 2 | 201190650 | 6.04536E-07 | *SPATS2L* | Spermatogenesis associated serine rich 2 like |
| rs159321 | 2q33.1 | 2 | 201184713 | 6.04536E-07 | *SPATS2L* | Spermatogenesis associated serine rich 2 like |
| rs34693942 | 2q33.1 | 2 | 201184348 | 6.04536E-07 | *SPATS2L* | Spermatogenesis associated serine rich 2 like |
| rs295142 | 2q33.1 | 2 | 201211716 | 6.63157E-07 | *SPATS2L* | Spermatogenesis associated serine rich 2 like |
| rs295117 | 2q33.1 | 2 | 201146828 | 6.68831E-07 | *SPATS2L* | Spermatogenesis associated serine rich 2 like |
| rs159320 | 2q33.1 | 2 | 201187775 | 6.78705E-07 | *SPATS2L* | Spermatogenesis associated serine rich 2 like |
| rs295136 | 2q33.1 | 2 | 201141002 | 7.00909E-07 | *SPATS2L* | Spermatogenesis associated serine rich 2 like |
| rs10882875 | 10q23.1 | 10 | 83023341 | 7.01825E-07 | *RPA2P2* | Replication protein A2 pseudogene 2 |
| rs1816541 | 2q33.1 | 2 | 201151011 | 7.07957E-07 | *SPATS2L* | Spermatogenesis associated serine rich 2 like |
| rs4672726 | 2q33.1 | 2 | 201149413 | 7.07957E-07 | *SPATS2L* | Spermatogenesis associated serine rich 2 like |
| rs11691757 | 2q33.1 | 2 | 201148951 | 7.07957E-07 | *SPATS2L* | Spermatogenesis associated serine rich 2 like |
| rs10497859 | 2q33.1 | 2 | 201144446 | 7.07957E-07 | *SPATS2L* | Spermatogenesis associated serine rich 2 like |
| rs4673891 | 2q33.1 | 2 | 201156362 | 7.21885E-07 | *SPATS2L* | Spermatogenesis associated serine rich 2 like |
| rs10931896 | 2q33.1 | 2 | 201148076 | 7.43016E-07 | *SPATS2L* | Spermatogenesis associated serine rich 2 like |
| rs12997220 | 2q33.1 | 2 | 201116111 | 7.88545E-07 | *SPATS2L* | Spermatogenesis associated serine rich 2 like |
| rs295118 | 2q33.1 | 2 | 201144004 | 8.0809E-07 | *SPATS2L* | Spermatogenesis associated serine rich 2 like |
| rs35467441 | 2q33.1 | 2 | 201106961 | 8.76943E-07 | *SPATS2L* | Spermatogenesis associated serine rich 2 like |
| rs4673912 | 2q33.1 | 2 | 201168993 | 9.63778E-07 | *SPATS2L* | Spermatogenesis associated serine rich 2 like |
| rs76358961 | 14q23.3 | 14 | 65659476 | 1.04768E-06 | *LINC02324* | Long intergenic non-protein coding RNA 2324 |
| rs7605146 | 2q33.1 | 2 | 201183888 | 1.07766E-06 | *SPATS2L* | Spermatogenesis associated serine rich 2 like |
| rs296801 | 2q33.1 | 2 | 201142812 | 1.20053E-06 | *SPATS2L* | Spermatogenesis associated serine rich 2 like |
| rs295140 | 2q33.1 | 2 | 201160699 | 1.25082E-06 | *SPATS2L* | Spermatogenesis associated serine rich 2 like |
| rs144092933 | 5q11.2 | 5 | 56224999 | 1.32515E-06 | *MIER3* | MIER family member 3 |
| rs56326533 | 2q33.1 | 2 | 201168758 | 1.37535E-06 | *SPATS2L* | Spermatogenesis associated serine rich 2 like |
| rs10931898 | 2q33.1 | 2 | 201171191 | 1.37535E-06 | *SPATS2L* | Spermatogenesis associated serine rich 2 like |
| rs3820888 | 2q33.1 | 2 | 201180023 | 1.37535E-06 | *SPATS2L* | Spermatogenesis associated serine rich 2 like |
| rs139060143 | 5q11.2 | 5 | 56239944 | 1.37751E-06 | *MIER3* | MIER family member 3 |
| rs4673904 | 2q33.1 | 2 | 201163454 | 1.40644E-06 | *SPATS2L* | Spermatogenesis associated serine rich 2 like |
| rs67414264 | 2q33.1 | 2 | 201206295 | 1.44831E-06 | *SPATS2L* | Spermatogenesis associated serine rich 2 like |
| rs11888462 | 2q33.1 | 2 | 201160499 | 1.63808E-06 | *SPATS2L* | Spermatogenesis associated serine rich 2 like |
| rs10931900 | 2q33.1 | 2 | 201177193 | 1.63808E-06 | *SPATS2L* | Spermatogenesis associated serine rich 2 like |
| rs7578220 | 2q33.1 | 2 | 201183701 | 1.63808E-06 | *SPATS2L* | Spermatogenesis associated serine rich 2 like |
| rs141893693 | 2q33.1 | 2 | 201179230 | 1.63808E-06 | *SPATS2L* | Spermatogenesis associated serine rich 2 like |
| rs7599609 | 2q33.1 | 2 | 201177653 | 1.63808E-06 | *SPATS2L* | Spermatogenesis associated serine rich 2 like |
| rs13028959 | 2q33.1 | 2 | 201185919 | 1.63808E-06 | *SPATS2L* | Spermatogenesis associated serine rich 2 like |
| rs4035396 | 2q33.1 | 2 | 201166460 | 1.63808E-06 | *SPATS2L* | Spermatogenesis associated serine rich 2 like |
| rs1367856 | 2q33.1 | 2 | 201160739 | 1.63808E-06 | *SPATS2L* | Spermatogenesis associated serine rich 2 like |
| rs7559005 | 2q33.1 | 2 | 201161371 | 1.63808E-06 | *SPATS2L* | Spermatogenesis associated serine rich 2 like |
| rs11696001 | 2q33.1 | 2 | 201162433 | 1.63808E-06 | *SPATS2L* | Spermatogenesis associated serine rich 2 like |
| rs71022314 | 2q33.1 | 2 | 201162264 | 1.63808E-06 | *SPATS2L* | Spermatogenesis associated serine rich 2 like |
| rs10931897 | 2q33.1 | 2 | 201162520 | 1.63808E-06 | *SPATS2L* | Spermatogenesis associated serine rich 2 like |
| rs67350115 | 2q33.1 | 2 | 201163234 | 1.63808E-06 | *SPATS2L* | Spermatogenesis associated serine rich 2 like |
| rs4516413 | 2q33.1 | 2 | 201163920 | 1.63808E-06 | *SPATS2L* | Spermatogenesis associated serine rich 2 like |
| rs4673906 | 2q33.1 | 2 | 201163812 | 1.63808E-06 | *SPATS2L* | Spermatogenesis associated serine rich 2 like |
| rs1367857 | 2q33.1 | 2 | 201160765 | 1.63808E-06 | *SPATS2L* | Spermatogenesis associated serine rich 2 like |
| rs13026151 | 2q33.1 | 2 | 201164570 | 1.63808E-06 | *SPATS2L* | Spermatogenesis associated serine rich 2 like |
| rs10931899 | 2q33.1 | 2 | 201176901 | 1.63808E-06 | *SPATS2L* | Spermatogenesis associated serine rich 2 like |
| rs7584810 | 2q33.1 | 2 | 201167772 | 1.63808E-06 | *SPATS2L* | Spermatogenesis associated serine rich 2 like |
| rs55677233 | 2q33.1 | 2 | 201168695 | 1.63808E-06 | *SPATS2L* | Spermatogenesis associated serine rich 2 like |
| rs12614621 | 2q33.1 | 2 | 201171207 | 1.63808E-06 | *SPATS2L* | Spermatogenesis associated serine rich 2 like |
| rs12618057 | 2q33.1 | 2 | 201171355 | 1.63808E-06 | *SPATS2L* | Spermatogenesis associated serine rich 2 like |
| rs3739121 | 2q33.1 | 2 | 201171014 | 1.63808E-06 | *SPATS2L* | Spermatogenesis associated serine rich 2 like |
| rs71022316 | 2q33.1 | 2 | 201171469 | 1.63808E-06 | *SPATS2L* | Spermatogenesis associated serine rich 2 like |
| rs67190025 | 2q33.1 | 2 | 201172086 | 1.63808E-06 | *SPATS2L* | Spermatogenesis associated serine rich 2 like |
| rs74853338 | 2q33.1 | 2 | 201171651 | 1.63808E-06 | *SPATS2L* | Spermatogenesis associated serine rich 2 like |
| rs7580924 | 2q33.1 | 2 | 201172628 | 1.63808E-06 | *SPATS2L* | Spermatogenesis associated serine rich 2 like |
| rs13005573 | 2q33.1 | 2 | 201164696 | 1.63808E-06 | *SPATS2L* | Spermatogenesis associated serine rich 2 like |
| rs1431766 | 2q33.1 | 2 | 201191034 | 1.63808E-06 | *SPATS2L* | Spermatogenesis associated serine rich 2 like |
| rs3769476 | 2q33.1 | 2 | 201181428 | 1.63808E-06 | *SPATS2L* | Spermatogenesis associated serine rich 2 like |
| rs35858375 | 2q33.1 | 2 | 201198623 | 1.66307E-06 | *SPATS2L* | Spermatogenesis associated serine rich 2 like |
| rs296790 | 2q33.1 | 2 | 201232862 | 1.74009E-06 | *SPATS2L* | Spermatogenesis associated serine rich 2 like |
| rs295132 | 2q33.1 | 2 | 201233502 | 1.74009E-06 | *SPATS2L* | Spermatogenesis associated serine rich 2 like |
| rs112170540 | 12q24.33 | 12 | 131912341 | 1.77668E-06 | *LOC101929974* | Uncharacterized LOC101929974 |
| rs295129 | 2q33.1 | 2 | 201229473 | 1.90752E-06 | *SPATS2L* | Spermatogenesis associated serine rich 2 like |
| rs11890234 | 2q33.1 | 2 | 201206706 | 1.95073E-06 | *SPATS3L* | Spermatogenesis associated serine rich 3 like |
| rs10459299 | 13q32.3 | 13 | 99776084 | 2.17179E-06 | *DOCK9-AS2* | DOCK9 antisense RNA 2 |
| rs77922030 | 5q31.3 | 5 | 141037683 | 2.30008E-06 | *ARAP3* | ArfGAP with RhoGAP domain, ankyrin repeat and PH domain 3 |
| rs35565614 | 2q33.1 | 2 | 201114245 | 2.49593E-06 | *SPATS2L* | Spermatogenesis associated serine rich 2 like |
| rs78037592 | 2q33.1 | 2 | 201105734 | 2.63252E-06 | *SPATS2L* | Spermatogenesis associated serine rich 2 like |
| rs2036013 | 8q23.3 | 8 | 116081494 | 2.69638E-06 | *LOC107986901* | Uncharacterized LOC107986901 |
| rs9463802 | 6p12.2 | 6 | 52469904 | 2.93425E-06 | *TRAM2-AS1* | TRAM2 antisense RNA 1 |
| rs12104649 | 2q33.1 | 2 | 201128832 | 2.95951E-06 | *SPATS2L* | Spermatogenesis associated serine rich 2 like |
| rs60004783 | 8q23.3 | 8 | 116078880 | 3.08532E-06 | *LOC107986901* | Uncharacterized LOC107986901 |
| rs6993473 | 8q23.3 | 8 | 116054890 | 3.08532E-06 | *LOC107986901* | Uncharacterized LOC107986901 |
| rs12548607 | 8q23.3 | 8 | 116062127 | 3.08532E-06 | *LOC107986901* | Uncharacterized LOC107986901 |
| rs62513383 | 8q23.3 | 8 | 116052133 | 3.08532E-06 | *LOC107986901* | Uncharacterized LOC107986901 |
| rs2132024 | 8q23.3 | 8 | 116051254 | 3.08532E-06 | *LOC107986901* | Uncharacterized LOC107986901 |
| rs12543621 | 8q23.3 | 8 | 116047935 | 3.08532E-06 | *LOC107986901* | Uncharacterized LOC107986901 |
| rs2306338 | 5q31.3 | 5 | 141039160 | 3.1582E-06 | *ARAP3* | ArfGAP with RhoGAP domain, ankyrin repeat and PH domain 3 |
| rs58787719 | 8q23.3 | 8 | 116083450 | 3.15969E-06 | *LOC107986901* | Uncharacterized LOC107986901 |
| rs11292682 | 2q33.1 | 2 | 201154388 | 3.16343E-06 | *SPATS2L* | Spermatogenesis associated serine rich 2 like |
| rs3734434 | 6p12.2 | 6 | 52460604 | 3.28912E-06 | *TRAM2-AS1* | TRAM2 antisense RNA 1 |
| 9:92462689 | 9q22.2 | 9 | 92462689 | 3.30062E-06 | *LOC100129066* | Uncharacterized LOC100129066 |
| rs10708262 | 2q33.1 | 2 | 201170509 | 3.30078E-06 | *SPATS2L* | Spermatogenesis associated serine rich 2 like |
| 8:116083355 | 8q23.3 | 8 | 116083355 | 3.34265E-06 | *LOC107986901* | Uncharacterized LOC107986901 |
| rs1353277 | 8q23.3 | 8 | 116079440 | 3.59954E-06 | *LOC107986901* | Uncharacterized LOC107986901 |
| rs958703 | 8q23.3 | 8 | 116080357 | 3.59954E-06 | *LOC107986901* | Uncharacterized LOC107986901 |
| rs57066356 | 8q23.3 | 8 | 116078926 | 3.59954E-06 | *LOC107986901* | Uncharacterized LOC107986901 |
| rs1472264 | 8q23.3 | 8 | 116065863 | 3.62359E-06 | *LOC107986901* | Uncharacterized LOC107986901 |
| rs7465536 | 8q23.3 | 8 | 116077043 | 3.62359E-06 | *LOC107986901* | Uncharacterized LOC107986901 |
| rs2357833 | 8q23.3 | 8 | 116074281 | 3.62359E-06 | *LOC107986901* | Uncharacterized LOC107986901 |
| rs7003216 | 8q23.3 | 8 | 116069481 | 3.62359E-06 | *LOC107986901* | Uncharacterized LOC107986901 |
| rs11997348 | 8q23.3 | 8 | 116083621 | 3.82216E-06 | *LOC107986901* | Uncharacterized LOC107986901 |
| rs1118828 | 8q23.3 | 8 | 116082778 | 3.82216E-06 | *LOC107986901* | Uncharacterized LOC107986901 |
| rs11189018 | 10q23.1 | 10 | 83018459 | 3.83118E-06 | *RPA2P2* | Replication protein A2 pseudogene 2 |
| rs11189019 | 10q23.1 | 10 | 83018925 | 3.83118E-06 | *RPA2P2* | Replication protein A2 pseudogene 2 |
| rs11189017 | 10q23.1 | 10 | 83018377 | 3.83118E-06 | *RPA2P2* | Replication protein A2 pseudogene 2 |
| rs10882870 | 10q23.1 | 10 | 83019949 | 3.83118E-06 | *RPA2P2* | Replication protein A2 pseudogene 2 |
| rs10509429 | 10q23.1 | 10 | 83018218 | 3.83118E-06 | *RPA2P2* | Replication protein A2 pseudogene 2 |
| 10:83018967 | 10q23.1 | 10 | 83018967 | 3.83118E-06 | *RPA2P2* | Replication protein A2 pseudogene 2 |
| rs112774911 | 10q23.1 | 10 | 83018963 | 3.83118E-06 | *RPA2P2* | Replication protein A2 pseudogene 2 |
| rs1898280 | 8q23.3 | 8 | 116074460 | 3.83141E-06 | *LOC107986901* | Uncharacterized LOC107986901 |
| rs7007151 | 8q23.3 | 8 | 116075541 | 4.22635E-06 | *LOC107986901* | Uncharacterized LOC107986901 |
| rs73355401 | 8q23.3 | 8 | 116076840 | 4.22635E-06 | *LOC107986901* | Uncharacterized LOC107986901 |
| rs7465541 | 8q23.3 | 8 | 116077115 | 4.22635E-06 | *LOC107986901* | Uncharacterized LOC107986901 |
| rs1494743 | 8q23.3 | 8 | 116075602 | 4.22635E-06 | *LOC107986901* | Uncharacterized LOC107986901 |
| rs60087678 | 2q33.1 | 2 | 201054083 | 4.47088E-06 | *SPATS2L* | Spermatogenesis associated serine rich 2 like |
| rs11308720 | 8q23.3 | 8 | 116078165 | 4.53294E-06 | *LOC107986901* | Uncharacterized LOC107986901 |
| rs55985986 | 2q33.1 | 2 | 201120987 | 4.6739E-06 | *SPATS2L* | Spermatogenesis associated serine rich 2 like |
| rs34840426 | 2q33.1 | 2 | 201084881 | 4.72305E-06 | *SPATS2L* | Spermatogenesis associated serine rich 2 like |
| rs79725795 | 2q33.1 | 2 | 201083534 | 4.72703E-06 | *SPATS2L* | Spermatogenesis associated serine rich 2 like |
| rs141447808 | 2q33.1 | 2 | 201083193 | 4.72703E-06 | *SPATS2L* | Spermatogenesis associated serine rich 2 like |
| rs17665563 | 6p12.2 | 6 | 52458872 | 4.8674E-06 | *TRAM2-AS1* | TRAM2 antisense RNA 1 |
| rs35030185 | 2q33.1 | 2 | 201108181 | 5.02773E-06 | *SPATS2L* | Spermatogenesis associated serine rich 2 like |
| rs144726176 | 6p12.2 | 6 | 52470750 | 5.06088E-06 | *TRAM2-AS1* | TRAM2 antisense RNA 1 |
| rs150355677 | 6p12.2 | 6 | 52470748 | 5.06088E-06 | *TRAM2-AS1* | TRAM2 antisense RNA 1 |
| rs148851872 | 6p12.2 | 6 | 52470746 | 5.06088E-06 | *TRAM2-AS1* | TRAM2 antisense RNA 1 |
| rs150665114 | 2q33.1 | 2 | 201073851 | 5.16868E-06 | *SPATS2L* | Spermatogenesis associated serine rich 2 like |
| rs1534599 | 2q33.1 | 2 | 201073133 | 5.28395E-06 | *SPATS3L* | Spermatogenesis associated serine rich 3 like |
| rs10104533 | 8q23.3 | 8 | 116082352 | 5.40907E-06 | *LOC107986901* | Uncharacterized LOC107986901 |
| rs34788019 | 2q33.1 | 2 | 201192126 | 5.44499E-06 | *SPATS2L* | Spermatogenesis associated serine rich 2 like |
| rs146896478 | 16q23.1 | 16 | 76480816 | 5.48406E-06 | *CNTNAP4* | Contactin associated protein like |
| rs35102285 | 2q33.1 | 2 | 201053255 | 5.54493E-06 | *LOC105373833* | Uncharacterized LOC105373833 |
| rs11998330 | 8q23.3 | 8 | 116084384 | 5.55332E-06 | *LOC107986901* | Uncharacterized LOC107986901 |
| rs11188915 | 10q23.1 | 10 | 82980696 | 5.56277E-06 | *RPA2P2* | Replication protein A2 pseudogene 2 |
| rs10882844 | 10q23.1 | 10 | 82982723 | 5.56277E-06 | *RPA2P2* | Replication protein A2 pseudogene 2 |
| rs17097396 | 10q23.1 | 10 | 82980927 | 5.56277E-06 | *RPA2P2* | Replication protein A2 pseudogene 2 |
| rs10882845 | 10q23.1 | 10 | 82982861 | 5.56277E-06 | *RPA2P2* | Replication protein A2 pseudogene 2 |
| rs12214270 | 6p12.2 | 6 | 52472466 | 5.63695E-06 | *TRAM2-AS1* | TRAM2 antisense RNA 1 |
| rs1653299 | 2q33.1 | 2 | 201075611 | 5.73202E-06 | *SPATS2L* | Spermatogenesis associated serine rich 2 like |
| rs755853 | 6p21.2 | 6 | 39140384 | 5.83855E-06 | *KCNK5* | Potassium two pore domain channel subfamily K member 5 |
| rs117348317 | 5p13.1 | 5 | 39847332 | 5.87419E-06 | *GCSHP1* | Glycine cleavage system protein H pseudogene 1 |
| rs6900718 | 6p12.2 | 6 | 52484391 | 5.8998E-06 | *TRAM2-AS1* | TRAM2 antisense RNA 1 |
| rs2749048 | 6p12.2 | 6 | 52483868 | 5.8998E-06 | *TRAM2-AS1* | TRAM2 antisense RNA 1 |
| rs144988506 | 2q33.1 | 2 | 201197495 | 6.31146E-06 | *SPATS2L* | Spermatogenesis associated serine rich 2 like |
| rs149384050 | 5p13.1 | 5 | 39753973 | 6.60535E-06 | *INTS6P1* | Integrator complex subunit 6 pseudogene 1 |
| rs11759608 | 6p12.2 | 6 | 52465706 | 6.66714E-06 | *TRAM2-AS1* | TRAM2 antisense RNA 1 |
| rs10931890 | 2q33.1 | 2 | 201102055 | 6.93855E-06 | *SPATS2L* | Spermatogenesis associated serine rich 2 like |
| rs11689664 | 2q33.1 | 2 | 201099961 | 6.93855E-06 | *SPATS2L* | Spermatogenesis associated serine rich 2 like |
| rs10931889 | 2q33.1 | 2 | 201101900 | 6.93855E-06 | *SPATS2L* | Spermatogenesis associated serine rich 2 like |
| rs76112266 | 2q33.1 | 2 | 201087157 | 6.93855E-06 | *SPATS2L* | Spermatogenesis associated serine rich 2 like |
| rs6995820 | 8p21.3 | 8 | 19180193 | 6.9834E-06 | *SH2D4A* | SH2 domain containing 4A |
| rs74751827 | 8p21.3 | 8 | 19179444 | 6.9834E-06 | *SH2D4A* | SH2 domain containing 4A |
| rs6739563 | 2q33.1 | 2 | 201134216 | 7.09522E-06 | *SPATS2L* | Spermatogenesis associated serine rich 2 like |
| rs12569444 | 10q23.1 | 10 | 83004750 | 7.28451E-06 | *RPA2P2* | Replication protein A2 pseudogene 2 |
| rs137873240 | 10q23.1 | 10 | 83004610 | 7.28451E-06 | *FARSBP1* | FARSB pseudogene 1 |
| rs76183688 | 10q23.1 | 10 | 82996766 | 7.40294E-06 | *RPA2P2* | Replication protein A2 pseudogene 2 |
| rs117310586 | 10q23.1 | 10 | 82999206 | 7.40294E-06 | *RPA2P2* | Replication protein A2 pseudogene 2 |
| rs149004734 | 10q23.1 | 10 | 82997250 | 7.40294E-06 | *RPA2P2* | Replication protein A2 pseudogene 2 |
| rs11188981 | 10q23.1 | 10 | 83001256 | 7.40294E-06 | *RPA2P2* | Replication protein A2 pseudogene 2 |
| rs10882860 | 10q23.1 | 10 | 82999681 | 7.40294E-06 | *RPA2P2* | Replication protein A2 pseudogene 2 |
| rs11528105 | 10q23.1 | 10 | 82995763 | 7.40294E-06 | *RPA2P2* | Replication protein A2 pseudogene 2 |
| rs913429 | 13q32.3 | 13 | 99775027 | 7.43756E-06 | *DOCK9-AS2* | DOCK9 divergent transcript |
| rs10445791 | 2q33.1 | 2 | 201078307 | 7.45039E-06 | *SPATS2L* | Spermatogenesis associated serine rich 2 like |
| rs10445792 | 2q33.1 | 2 | 201078821 | 7.45039E-06 | *SPATS2L* | Spermatogenesis associated serine rich 2 like |
| rs78736401 | 10q23.1 | 10 | 82985461 | 7.46591E-06 | *RPA2P2* | Replication protein A2 pseudogene 2 |
| rs295139 | 2q33.1 | 2 | 201159988 | 7.63326E-06 | *SPATS4L* | Spermatogenesis associated serine rich 4 like |
| rs9470990 | 6p21.2 | 6 | 39137027 | 7.92437E-06 | *KCNK5* | Potassium two pore domain channel subfamily K member 5 |
| rs9951646 | 18q23 | 18 | 77374884 | 8.32731E-06 | *LOC105372228* | Uncharacterized LOC105372228 |
| rs35350286 | 6p12.2 | 6 | 52456432 | 8.38179E-06 | *TRAM2-AS1* | TRAM2 antisense RNA 1 |
| rs67706850 | 6p12.2 | 6 | 52456776 | 8.38179E-06 | *TRAM2-AS1* | TRAM2 antisense RNA 1 |
| rs2561412 | 6p21.2 | 6 | 39132981 | 8.57397E-06 | *KCNK5* | Potassium two pore domain channel subfamily K member 5 |
| rs10947785 | 6p21.2 | 6 | 39132818 | 8.57397E-06 | *KCNK5* | Potassium two pore domain channel subfamily K member 5 |
| rs74954137 | 14q31.3 | 14 | 85841672 | 8.62652E-06 | *LINC02329* | Long intergenic non-protein coding RNA 2329 |
| 2:201073861 | 2q33.1 | 2 | 201073861 | 8.87271E-06 | *SPATS2L* | Spermatogenesis associated serine rich 2 like |
| rs17208495 | 5q31.3 | 5 | 141043268 | 9.12135E-06 | *ARAP3* | ArfGAP with RhoGAP domain, ankyrin repeat and PH domain 3 |
| rs17097438 | 5q31.3 | 5 | 141046936 | 9.4747E-06 | *ARAP3* | ArfGAP with RhoGAP domain, ankyrin repeat and PH domain 3 |
| rs34188961 | 11q11 | 11 | 55421471 | 9.62409E-06 | *OR4S2* | Olfactory receptor family 4 subfamily S member 2 |
| rs13256716 | 8p21.3 | 8 | 23007021 | 9.68245E-06 | *TNFRSF10D* | TNF receptor superfamily member 10d |
| rs79897806 | 2q33.1 | 2 | 201073968 | 9.94087E-06 | *SPATS2L* | Spermatogenesis associated serine rich 2 like |
| rs112486506 | 2q33.1 | 2 | 201073947 | 9.94087E-06 | *SPATS2L* | Spermatogenesis associated serine rich 2 like |

**Note**: CHR, chromosome; BP, base pair
