# Supplementary material for: Heritability and genome-wide association analyses of fasting plasma glucose in Chinese adult twins
Source: BMC Genomics. 2020 Jul 18;21:491. doi: 10.1186/s12864-020-06898-z (PMC7368793; doi:10.1186/s12864-020-06898-z)
Supplement: Supplementary file 4 — Additional file 4. Comparison between our imputed SNPs (P-value < 0.05) and other previously reported SNPs in GWAS. [file 12864_2020_6898_MOESM4_ESM.docx]

| **SNP** | **Chr** | **Genes** | **Our study** | |  | **Other studies** | | | | | |
| --- | --- | --- | --- | --- | --- | --- | --- | --- | --- | --- | --- |
|  |  |  | Beta | *P*-value |  | Reference | Population | Samples size | Trait | Beta | *P*-value |
| rs7684538 | 4 | *UNC5C* | 0.4331161 | 3.75E-02 |  | Meigs, et al. | American | 1087 | Fasting plasma glucose | NA | 5.00E-05 |
| rs2367204 | 2 | *IMMT* | 0.2480871 | 4.44E-02 |  | Meigs, et al. | American | 1087 | Fasting plasma glucose | NA | 7.00E-03 |
| rs7186570 | 16 | *A2BP1* | -0.2002955 | 3.74E-02 |  | Meigs, et al. | American | 1087 | Fasting plasma glucose | NA | 1.00E-02 |
| rs861085 | 5 | *NUDT12* | 0.4105807 | 2.93E-02 |  | Meigs, et al. | American | 1087 | Fasting plasma glucose | NA | 8.00E-03 |
| rs1402837 | 2 | *G6PC2* | 0.1972862 | 4.03E-02 |  | Chambers, et al. | Indian Asian | 5089 | Fasting plasma glucose | NA | 2.00E-08 |
| rs2302593 | 19 | *GIPR* | -0.2000784 | 3.08E-02 |  | Ivarsdottir, et al. | Icelander | 117548 | Fasting plasma glucose | 0.025 | 1.40E-04 |
| rs4869272 | 5 | *PCSK1* | -0.2242843 | 1.77E-02 |  | Ivarsdottir, et al. | Icelander | 117548 | Fasting plasma glucose | 0.022 | 1.90E-03 |
| rs492594 | 2 | *G6PC2* | -0.1942456 | 3.70E-02 |  | Spracklen, et al. | Chinese | 8045 | Fasting plasma glucose | -0.059 | 2.00E-03 |

**Additional file 4.** Comparison between our imputed SNPs (*P*-value < 0.05) and other previously reported SNPs in GWAS

**Note**: NA, not available
