# Supplementary material for: Heritability and genome-wide association analyses of fasting plasma glucose in Chinese adult twins
Source: BMC Genomics. 2020 Jul 18;21:491. doi: 10.1186/s12864-020-06898-z (PMC7368793; doi:10.1186/s12864-020-06898-z)
Supplement: Supplementary file 5 — Additional file 5. The characteristics of subjects in validation analysis. [file 12864_2020_6898_MOESM5_ESM.docx]

**Additional file 5**. The characteristics of subjects in validating analysis

| **Group** | **Gender** | **Age, years** | **BMI, kg/m^2^** | **FPG, mmol/L** |
| --- | --- | --- | --- | --- |
| Case | Female | 48 | 29.2 | 6.8 |
| Case | Male | 52 | 25.7 | 6.1 |
| Case | Male | 63 | 19.3 | 6.8 |
| Case | Male | 63 | 24.6 | 7.9 |
| Control | Female | 53 | 34.1 | 4.66 |
| Control | Female | 53 | 27.5 | 4.31 |
| Control | Male | 47 | 22.2 | 4.6 |
| Control | Male | 59 | 20.2 | 4.5 |

**Note**: the cases were defined as impaired fasting glucose (IFG) status, i.e. fasting plasma glucose (FPG)≥6.1 mmol/L
