# Supplementary material for: Heritability and genome-wide association analyses of fasting plasma glucose in Chinese adult twins
Source: BMC Genomics. 2020 Jul 18;21:491. doi: 10.1186/s12864-020-06898-z (PMC7368793; doi:10.1186/s12864-020-06898-z)
Supplement: Supplementary file 6 — Additional file 6. The questionare of survey on health status of elderly twins in Qingdao city. [file 12864_2020_6898_MOESM6_ESM.doc]

| Questionnaire No. | □□□□ |
| --- | --- |

**Survey on Health Status of Elderly Twins in Qingdao City**

**
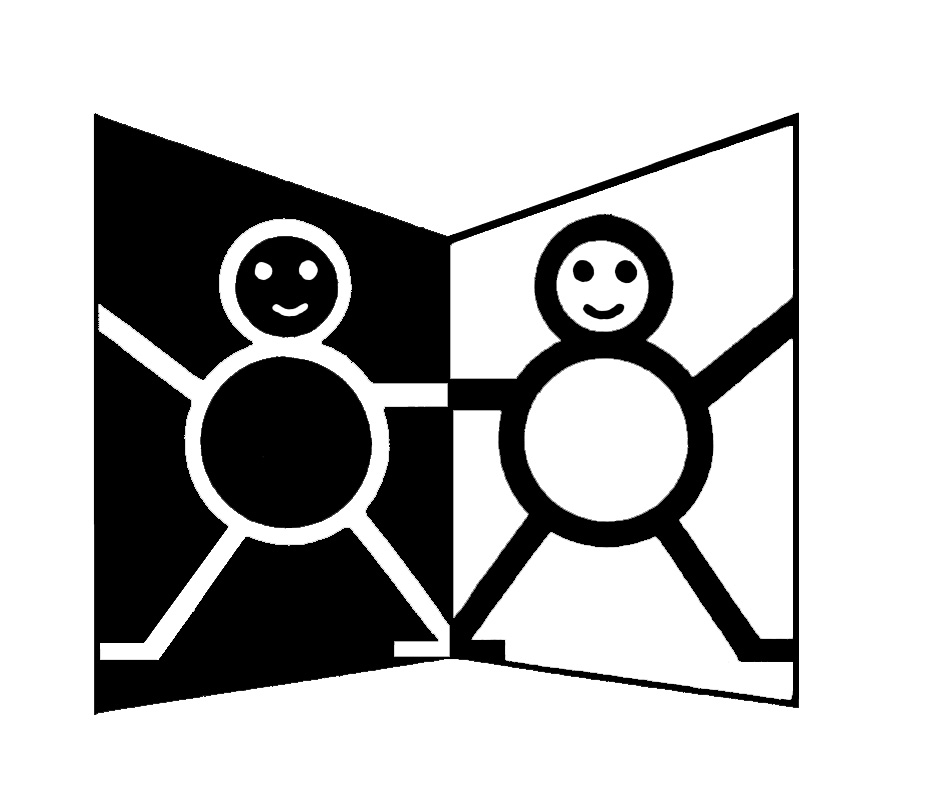
**

**Qingdao Municipal Center for Disease Control and Prevention Qingdao Municipal Twins Health Promotion Association**

| **Part 1: The basic information** |
| --- |

Name of twins：__________

Gender: 1. male 2. female

Id No.：

Date of birth：_______year_______month Place of birth：________ province________ county

Have you ever participated in the medical examination of twins？1. yes，I did in the year of 2. no

Home address： Telephone: __________

Work address： Telephone: __________

**A．Twins in general condition**

| 1. | Are you of the same sex? |  |  |
| --- | --- | --- | --- |
|  | □1 yes |  |  |
|  | □2 no |  |  |

**B．Education, marital and work condition**

| 2. | What is your education level? |  | N18N |
| --- | --- | --- | --- |
|  | □1 have never been to school |  |  |
|  | □2 primary school |  |  |
|  | □3 junior middle school |  |  |
|  | □4 high school |  |  |
|  | □5 college degree or above |  |  |

| 3. | What is your marital status? |  | N18N |
| --- | --- | --- | --- |
|  | □1 married |  |  |
|  | □2 remarried |  |  |
|  | □3 unmarried |  |  |
|  | □4 separated |  |  |
|  | □5 divorce |  |  |
|  | □6 widowed |  |  |

| 4. | Who are you living with now? | | |  |
| --- | --- | --- | --- | --- |
|  | □1 family |  |  |  |
|  | □2 living alone（skip to question 6） |  |  |
|  | □3 nursing home（skip to question 6） |  |  |

| 5. | How many people (excluding yourself) are you living with? | *23 |
| --- | --- | --- |
|  | number： __________ |  |

| 6. | How many years have you lived alone or in a nursing home? | *23 |
| --- | --- | --- |
|  | number： _________ |  |

| 7. | | Are you still working? | |  | |
| --- | --- | --- | --- | --- | --- |
|  | | □1 yes, full time (8 hours or more every day) | |  |  |
|  | | □2 yes, part-time (under 8 hours every day) | |  |  |
|  | | □3 no, there is disability gold |  |  | since |
|  | | □4 no, there is retirement |  | year： |  |
|  | | □5 no, there is a pension |  |  |  |
|  | | □6 prolonged illness exceeding 14 days | |  |  |
|  | □7 others __________________________________ | | |  |  |

| 8. | What was your last job? | *23 |
| --- | --- | --- |
|  | career： ___________________________ |  |

| **Part 2: Body functions** |
| --- |

1. **Health status**

| 9. | What do you think of your own health in general? | *18 |
| --- | --- | --- |
|  | □1 very good |  |
|  | □2 good |  |
|  | □3 general |  |
|  | □4 poor |  |
|  | □5 very poor |  |

| 10. | **How do you think of your health comparing with your twin brother (sister)**? |  |
| --- | --- | --- |
|  | □1 better |  |
|  | □2 the same |  |
|  | □3 poorer |  |
|  | □4 don't know |  |

| 11. | **How do you think of your health comparing with your peers**? |  |
| --- | --- | --- |
|  | □1 better |  |
|  | □2 the same |  |
|  | □3 poorer |  |
|  | □4 don't know |  |

| 12. | **Can you do what you want to do**? |  |
| --- | --- | --- |
|  | □1 yes, always |  |
|  | □2 yes, most of the time |  |
|  | □3 yes, occasionally |  |
|  | □4 no, it's almost difficult |  |
|  | □5 no, never |  |

| 13. | **Have you ever or are you informed by your doctor that you have any of the following diseases**? | | | | |
| --- | --- | --- | --- | --- | --- |
| disease | | Yes, now | Yes, ever | Never | The age of being diagnosed firstly |
| 01．Hypertension | | □1 | □2 | □3 | *25s |
| 02．Diabetes | | □1 | □2 | □3 |  |
| 03．Heart disease | | □1 | □2 | □3 |  |
| 04．Stroke and cerebrovascular disease | | □1 | □2 | □3 |  |
| 05．Bronchitis, emphysema, asthma or pneumonia | | □1 | □2 | □3 |  |
| 06．Tuberculosis | | □1 | □2 | □3 |  |
| 07．Cataract | | □1 | □2 | □3 | *25o |
| 08．Glaucoma | | □1 | □2 | □3 |  |
| 09．Cancer | | □1 | □2 | □3 |  |
| 10．Prostate disease | | □1 | □2 | □3 |  |
| 11．Gastrointestinal ulcer | | □1 | □2 | □3 |  |
| 12．Parkinson's disease | | □1 | □2 | □3 |  |
| 13．Pressure ulcers | | □1 | □2 | □3 |  |
| 14．Arthritis | | □1 | □2 | □3 |  |
| 15．Dementia | | □1 | □2 | □3 |  |
| 16. Epilepsy | | □1 | □2 | □3 |  |
| 17. Cholecystitis or cholelithiasis | | □1 | □2 | □3 |  |
| 18. Dyslipidemia | | □1 | □2 | □3 |  |
| 19. Rheumatism or rheumatoid arthritis | | □1 | □2 | □3 |  |
| 20. Chronic nephritis | | □1 | □2 | □3 |  |
| 21. Hyperplasia of mammary gland | | □1 | □2 | □3 |  |
| 22. Uterine fibroids | | □1 | □2 | □3 |  |
| 23. Prostatic hyperplasia | | □1 | □2 | □3 |  |
| 24．Hepatitis | | □1 | □2 | □3 |  |
| 25. Others, please specify | | □1 | □2 | □3 |  |

**14~16** **Female responses and male skips answering**

| 14. | **What is your age of menarche**? |  |  |
| --- | --- | --- | --- |
|  | _________years |  |  |

| 15. | **Is your menstrual cycle normal**? |  |  |
| --- | --- | --- | --- |
|  | □1 yes，the menstrual cycle is days |  |  |
|  | □2 no |  |  |

| 16. | **Are you menopausal now**? |  |  |
| --- | --- | --- | --- |
|  | □1 yes， the age of amenorrhea was years |  |  |
|  | □2 no |  |  |

| **Part 5:**  **living habits** |
| --- |

**A．Smoking**

| 21. | **Do you smoke now**? |  |
| --- | --- | --- |
|  | □1 yes（skip to question 24） |  |
|  | □2 no |  |

| 22. | **Did you use to smoke**? |  | |
| --- | --- | --- | --- |
|  | □1 yes |  | |
|  | □2 no（skip to question 27） | |  |

| 23. | **How old were you when you quitted smoking**? |  |
| --- | --- | --- |
|  | age： ___________ |  |

| 24. | **How many years have you been smoking regularly**? |  |
| --- | --- | --- |
|  | years： ___________ |  |

| 25. | **Do you smoke by ingestion**? | medicin |
| --- | --- | --- |
|  | □1 yes |  |
|  | □2 no |  |

| 26. | **How many cigarettes do you smoke per day on average**? |  |
| --- | --- | --- |
|  | average number of cigarettes per day______ |  |

| 27. | **How many days in the past seven days (or weeks) have you been in the same room or transportation (bus, car, boat, or train) with someone who is smoking**? |  | N18N |
| --- | --- | --- | --- |
|  | □1 0 days |  |  |
|  | □2 1 days |  |  |
|  | □3 2 days |  |  |
|  | □4 3 days |  |  |
|  | □5 4 days |  |  |
|  | □6 5 days |  |  |
|  | □7 6 days |  |  |
|  | □8 7 days |  |  |
|  | □9 don't know |  |  |

**B.** **Drinking (last six months)**

| 28. | **Are you drinking alcohol now**? |  |
| --- | --- | --- |
|  | □1 yes（skip to question 31） |  |
|  | □2 no |  |

| 29. | **Have you ever drunk alcohol in the past**? |  |
| --- | --- | --- |
|  | □1 yes |  |
|  | □2 no（skip to question 34） |  |

| 30. | **At what age did you quit drinking**? |  |
| --- | --- | --- |
|  | age： ___________ |  |

| 31. | **How many years have you been drinking regularly**? |  |
| --- | --- | --- |
|  | years： ___________ |  |

| 32. | **What kind of wine do you drink**? （**Single option**） | medicin |
| --- | --- | --- |
|  | □1 high liquor（38^。^Or above)） |  |
|  | □2 low-alcohol white wine（below 38^。^） |  |
|  | □3 fruit wine |  |
|  | □4 rice wine |  |
|  | □5 beer |  |
|  | □6 others |  |

| 33. | **How many drinks do you (or did you) drink on average every day**? |  |
| --- | --- | --- |
|  | average amount of drinking per day ___ ___ |  |

**C．** **Eating habits**

| 34. | **How many glasses of water do you drink on average every day**? |  |
| --- | --- | --- |
|  | ______cups |  |

| 35. | **Do you have the habit of drinking tea**? |  |
| --- | --- | --- |
|  | □1 yes, there is a tea habit of ______ year |  |
|  | □2 no |  |

| 36. | **How often do you eat**： | | | | | | | | |
| --- | --- | --- | --- | --- | --- | --- | --- | --- | --- |
|  |  | Never | once a month or less | twice a month | once a week | once a week 2-3 times | once a day | 2-3 times a day | more than 4 times a day |
| 1) fruit | | □0 | □1 | □2 | □3 | □4 | □5 | □6 | □7 |
| 2) vegetables | | □0 | □1 | □2 | □3 | □4 | □5 | □6 | □7 |
| 3) fish and other aquatic products | | □0 | □1 | □2 | □3 | □4 | □5 | □6 | □7 |
| 4) soy products | | □0 | □1 | □2 | □3 | □4 | □5 | □6 | □7 |
| 5) dairy products | | □0 | □1 | □2 | □3 | □4 | □5 | □6 | □7 |
| 6) fried food | | □0 | □1 | □2 | □3 | □4 | □5 | □6 | □7 |
| 7) pickled pickles or pickles | | □0 | □1 | □2 | □3 | □4 | □5 | □6 | □7 |

**D．Physical activity and exercise (IPAQ)**

|  | **In the past 7 days, you have had several:** |  |  | | *18 |
| --- | --- | --- | --- | --- | --- |
|  |  | days | | the average time you spend doing these activities each day | never |
| **37.** | Do heavy physical activities, such as lifting (lifting) heavy objects, chopping wood, sweeping snow, mopping the floor, running, swimming, jumping and aerobics in the gym？ | days | | hours  minutes | □ |
| **38.** | Engage in moderate physical activities, such as carrying (lifting) light objects, sweeping the floor, cleaning Windows, cleaning the room, cooking, washing clothes, cycling, table tennis, badminton, ballroom dancing, etc.? Don't include walking | days | | hours  minutes | □ |
| **39.** | Walk for at least 10 minutes each time? This includes walking at work and at home, walking for transportation, and walking for exercise | days | | hours  minutes | *□* |
| **40.** | The final question is about the amount of time you spend sitting, whether at work or at home, at your desk, at your computer, sitting or lying down watching TV, visiting friends, reading, driving, etc. Over the past seven days, you have been sitting for about an hour each day | days | | hours  minutes |  |

**E．Cultural and entertainment activities (last year)**

| 41. | **Do you often read newspapers**？ |  |
| --- | --- | --- |
|  | □1 never |  |
|  | □2 once a year at least |  |
|  | □3 once a month at least |  |
|  | □4 once a week at least |  |
|  | □5 everyday |  |

| 42. | **Do you often read books or magazines**? |  |
| --- | --- | --- |
|  | □1 never |  |
|  | □2 once a year at least |  |
|  | □3 once a month at least |  |
|  | □4 once a week at least |  |
|  | □5 everyday |  |

| 43. | **Do you often write articles or keep a diary**? |  |
| --- | --- | --- |
|  | □1 never |  |
|  | □2 once a year at least |  |
|  | □3 once a month at least |  |
|  | □4 once a week at least |  |
|  | □5 everyday |  |

| 44. | **Do you often watch TV**? |  |
| --- | --- | --- |
|  | □1 never |  |
|  | □2 once a year at least |  |
|  | □3 once a month at least |  |
|  | □4 once a week at least |  |
|  | □5 everyday |  |

| 45. | **Do you travel a lot**? |  |
| --- | --- | --- |
|  | □1 never |  |
|  | □2 very few |  |
|  | □3 once every two years |  |
|  | □4 once a year |  |
|  | □5 many times a year |  |

| 46. | **Do you often go to the theatre, cinema or concerts**? |  |
| --- | --- | --- |
|  | □1 never |  |
|  | □2 once a year at least |  |
|  | □3 once a month at least |  |
|  | □4 once a week at least |  |
|  | □5 everyday |  |

| 47. | **Do you often use the internet or libraries to get information and solve problems**? |  |
| --- | --- | --- |
|  | □1 never |  |
|  | □2 once a year at least |  |
|  | □3 once a month at least |  |
|  | □4 once a week at least |  |
|  | □5 everyday |  |

**F．Social activities (last year)**

| 48. | **Do you often take part in social activities**? |  |
| --- | --- | --- |
|  | □1 never |  |
|  | □2 once a year at least |  |
|  | □3 once a month at least |  |
|  | □4 once a week at least |  |
|  | □5 everyday |  |

| 49. | **Do you often have dinner or get-together with your family and friends (including playing chess, mahjong, CARDS, etc.)?** |  |
| --- | --- | --- |
|  | □1 never |  |
|  | □2 once a year at least |  |
|  | □3 once a month at least |  |
|  | □4 once a week at least |  |
|  | □5 everyday |  |

| 50. | **Do you often contact your relatives and friends by phone or by letter**? |  |
| --- | --- | --- |
|  | □1 never |  |
|  | □2 once a year at least |  |
|  | □3 once a month at least |  |
|  | □4 once a week at least |  |
|  | □5 everyday |  |

| 51. | **Do you often entertain relatives and friends at home**? |  |
| --- | --- | --- |
|  | □1 never |  |
|  | □2 once a year at least |  |
|  | □3 once a month at least |  |
|  | □4 once a week at least |  |
|  | □5 everyday |  |

| 52. | **Do you often visit your relatives and friends**? |  |
| --- | --- | --- |
|  | □1 never |  |
|  | □2 once a year at least |  |
|  | □3 once a month at least |  |
|  | □4 once a week at least |  |
|  | □5 everyday |  |

| 53. | **Do you often see your twin brother (sister)?** |  |
| --- | --- | --- |
|  | □1 never |  |
|  | □2 once a year at least |  |
|  | □3 once a month at least |  |
|  | □4 once a week at least |  |
|  | □5 everyday |  |

| 54. | **Do you often keep in touch with your twin brother (sister) by phone**? |  |
| --- | --- | --- |
|  | □1 never |  |
|  | □2 once a year at least |  |
|  | □3 once a month at least |  |
|  | □4 once a week at least |  |
|  | □5 everyday |  |

**Physical examination**

| height __ __ __.__ __ cm weight __ __ __.__ __ kg BMI__ __ __.__ __  body fat rate __ __ __.__ __%  chest __ __ cm waist circumference __ __ cm hip circumference __ __ cm  blood pressure（mmHg） systolic diastolic  The first time __ __ __ __ __ __  The second time __ __ __ __ __ __  The third time __ __ __ __ __ __ |
| --- |
| lung capacity  the first time the second time  the first second lung capacity（FEV1） __ __ __ L __ __ __L  maximum lung capacity（FVC） __ __ __ L __ __ __L |
| grip strength   | the right hand： | 1. __ __ __kg | 2. __ __ __kg | 3. __ __ __kg | | --- | --- | --- | --- | | the left hand： | 1. __ __ __kg | 2. __ __ __kg | 3. __ __ __kg | |  |  |  |  | |
| leg strength   | Were the participants able to stand for five times？ |  | | --- | --- | | □1 yes |  | | □2 no, only 1-4 finishes |  | | □3 no, not even once |  | | the number of times_______ |  |  | results： |  | | --- | --- | | the number of pulse before the investigation： __ __ __ times/ minute |  | | the number of pulse after the investigation： __ __ __times/ minute |  | | time to complete 5 stands： __ __ seconds |  | |  |  | |  |  | |
| eye test  eyesight left eye right eye  far __ __ __ __  close __ __ __ __ |
| oral examination missing teeth ；  the first tooth falls out naturally (non-traumatic, tooth extraction and other external causes) time： year month |
| fundus image pasting place |
| listening:   |  | 0.5 Hz | 1 KHz | 2 KHz | 4 KHz | 8 KHz | 12.5 KHz | | --- | --- | --- | --- | --- | --- | --- | | left ear（dB） |  |  |  |  |  |  | | right ear（dB） |  |  |  |  |  |  | |
| abdomen B ultrasound pasting place |
| ankle blood pressure：  systolic blood pressure __ __ |
| blood routine, blood biochemical paste  blood type__ __ |

investigators： survey date： year month day

**Thank you again for your cooperation！**
